# Delta-8 Tetrahydrocannabinol Product Impurities

**DOI:** 10.3390/molecules27206924

**Published:** 2022-10-15

**Authors:** Colleen L. Ray, Madison P. Bylo, Jonny Pescaglia, James A. Gawenis, C. Michael Greenlief

**Affiliations:** 1 Department of Chemistry, University of Missouri, 601 S. College Avenue, Columbia, MO 65211, USA; 2 Sweetwater Science Laboratories, Glasgow, MO 65264, USA

**Keywords:** NMR, tetrahydrocannabinol, cannabis, hemp, cannabidiol, product safety

## Abstract

Due to increased concerns regarding unidentified impurities in delta-8 tetrahydrocannabinol (Δ-8 THC) consumer products, a study using Nuclear Magnetic Resonance (NMR), high performance liquid chromatography (HPLC), and mass spectrometry (MS) was conducted to further investigate these products. Ten Δ-8 THC products, including distillates and ready to use vaporizer cartridges, were analyzed. The results yield findings that the tested products contain several impurities in concentrations far beyond what is declared on certificates of analysis for these products. As Δ-8 THC is a synthetic product synthesized from cannabidiol (CBD), there are valid concerns regarding the presence of impurities in these products with unknown effects on the human body. Compounding this problem is apparent inadequate testing of these products by producers and independent laboratories.

## 1. Introduction

**Figure 1:**
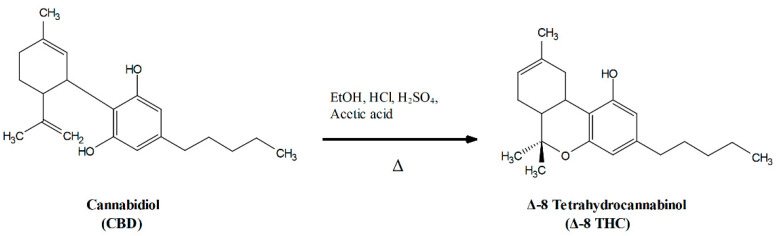
Example reaction scheme for one published synthesis of Δ-8 THC from CBD [3]. Inter-ring bond length on cannabidiol exaggerated for clarity.

Delta-8 tetrahydrocannabinol (Δ-8 THC) is a structural isomer of the well-known active ingredient in cannabis products, Δ-9 THC, differing only by the location of an unsaturated bond. Due to a technicality in the legal definition, hemp-derived Δ-8 THC became effectively unregulated by federal law in the United States as part of Section 10113 in The Agricultural Improvement Act of 2018 [1]. This has resulted in a number of hemp product producers marketing these products in regions where local laws do not address them by using CBD derived from industrial hemp as feedstock for this synthesis. Δ-8 THC is synthesized from CBD by a ring closure reaction often involving harsh reaction conditions (Figure 1) [2]. As with many organic syntheses, it is prone to side reactions. A recent mass spectrometry-based analysis of (Δ-8 THC) products reported finding a number of unknown impurities in these products [3].

Vaporizer cartridges and distillates are often sold with certificates of analysis (COA) to serve as a guide to the consumer about product purity. However, the methods of analysis are sometimes missing in the COA.

In order to further investigate potential unknowns in these products, a study was performed by analysis of ten Δ-8 THC products, of which half were sold as high purity distillates and the remainder were ready to use cartridges for use in vaporizer devices. The goal is to develop an NMR-based method of analysis that can quickly screen commercial Δ-8 THC products for the presence of possible contaminants. In the process of testing commercial products, we found a number of species not listed on certificate of analysis. Here, we report the combination of Nuclear Magnetic Resonance (NMR), high performance liquid chromatography (HPLC), and mass spectrometry (MS) to investigate the content of these commercial products.

## 2. Results and Discussion

**Figure 2:**
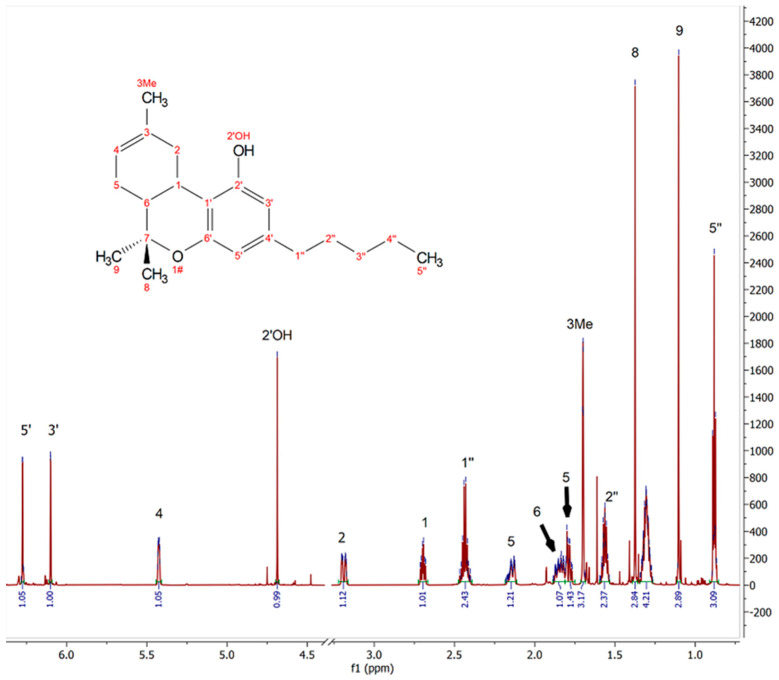
800 MHz ^1^H NMR spectrum of selected sample 3 (empty region from 3.35 ppm to 4.55 ppm omitted) (800 MHz, CDCl_3_) δ 6.27 (d, *J* = 1.6 Hz, 1H), 6.10 (d, *J* = 1.6 Hz, 1H), 5.49 – 5.37 (m, 1H), 4.67 (s, 1H), 3.19 (ddd, *J* = 17.6, 5.0, 1.9 Hz, 1H), 2.70 (td, *J* = 10.9, 4.8 Hz, 1H), 2.53 – 2.37 (m, 3H), 2.14 (ddt, *J* = 15.7, 4.3, 1.7 Hz, 1H), 1.89 – 1.81 (m, 1H), 1.80 – 1.75 (m, 1H), 1.70 (d, *J* = 1.2 Hz, 2H), 1.58–1.53 (m, 3H), 1.37 (s, 3H), 1.34 – 1.26 (m, 4H), 1.10 (s, 3H), 0.88 (t, *J* = 7.0 Hz, 3H).

Initial NMR analysis of the major peaks in the collected spectra show that the major product is consistent with published chemical shifts of Δ-8 THC [4] (Figure 2). With the integral for the proton signal on carbon 5′ calibrated to 1.0 proton, few of the other integrals match up to the expected values. As a result, the overall proton count by integration is 31 for Figure 2. A proton integration of 27 is expected for Δ-8 THC. While the deviations are slight, typically less than 10% deviation is expected, examination of the minor peaks show the presence of many impurities.

**Figure 3:**
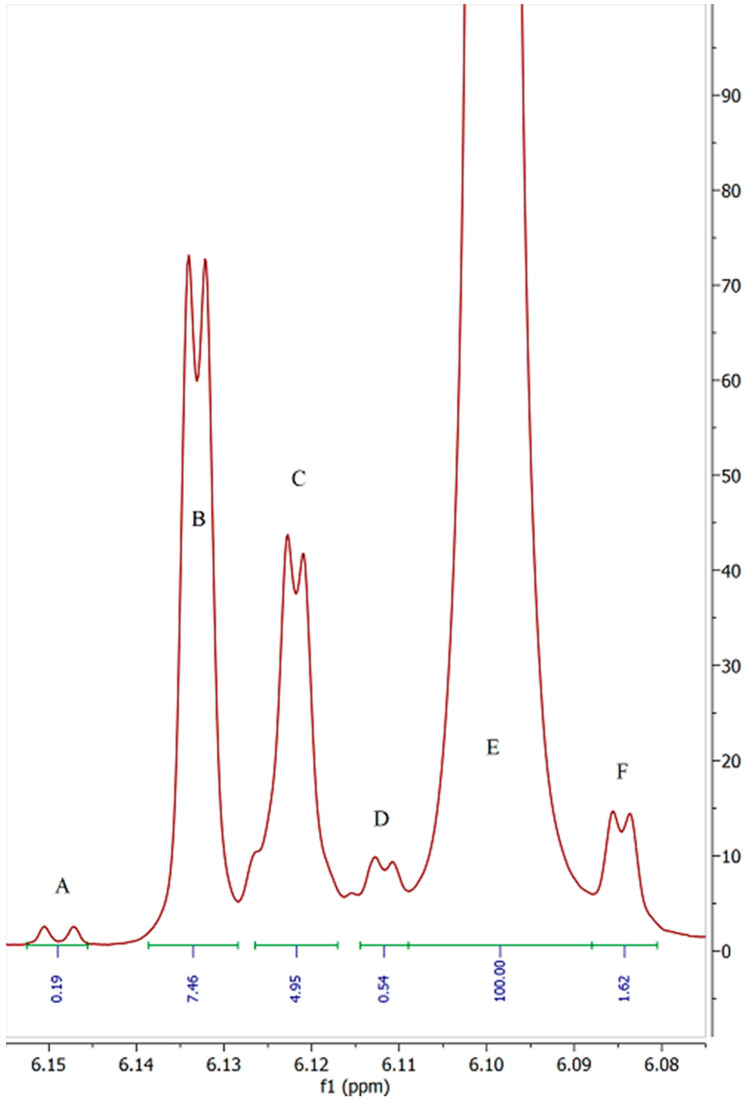
6.075 to 6.155 ppm region detailing 3**′** proton resonance of sample 3. ^1^H NMR (800 MHz, CDCl_3_) δ 6.15 (d, *J* = 2.7 Hz), 6.13 (d, *J* = 1.6 Hz), 6.12 (d, *J* = 1.6 Hz), 6.11 (d, *J* = 1.7 Hz), 6.10 (d, *J* = 1.6 Hz), 6.08 (d, *J* = 1.7 Hz). The labeled peaks are discussed in the text and correspond to the values shown in Table 1.

Upon closer inspection of the region surrounding the peak from the proton on carbon 5′ in sample 3 it becomes very apparent that there are multiple products in this sample (Figure 3). This sample was certified as 93.43% pure Δ-8 THC with no other cannabinoids detected.

**Figure 4:**
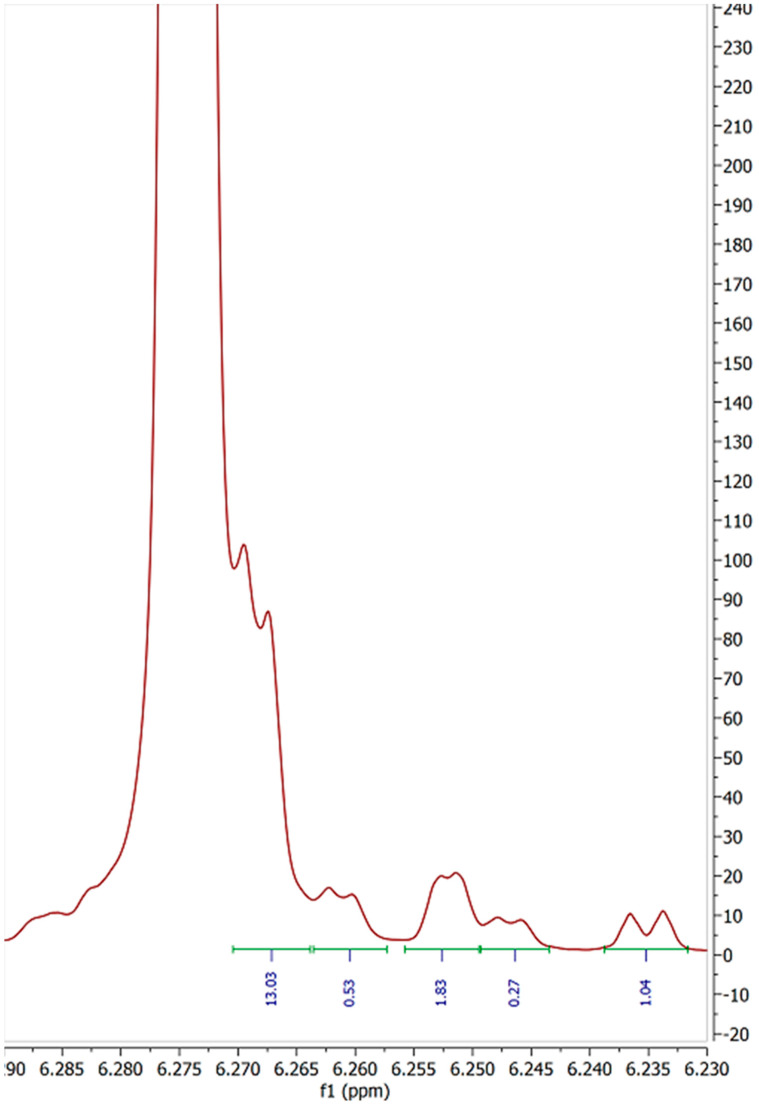
6.23 to 6.29 ppm region detailing 5**′** proton resonance of sample 3. (800 MHz, CDCl_3_) δ 6.15 (d, *J* = 2.4 Hz), 6.26 (d, *J* = 1.6 Hz), 6.25 (d, *J* = 1.4 Hz), 6.24 (d, *J* = 2.2 Hz).

The presence of four extraneous doublet peaks, all with J values of 1.6–1.7 Hz suggests that these peaks may well be equivalent protons on isomers of Δ-8 THC or some other form of cannabinoid. All other samples show similar impurities with the expanded spectral excerpts available in the Supplementary Materials (Sample S3). Peaks “A” and “D” in Figure 3 may potentially belong to compounds 1 and 2 described by Radwan [5]. Though the relatively congested peak area for carbon 3**′** shows no clear peak of similar integral value at 6.27 ppm and 6.24 ppm, respectively (Figure 4).

When the integral of the major product peak “E” (Figure 3) is calibrated to 100 protons it becomes quite clear that over 15% of this product is not consistent with Δ-8 THC, even when ignoring the extraneous peaks in the lower frequency portions of the spectrum. Table 1 details the relative impurity totals for all samples examined.

With the Δ-8 THC 5**′** peak (peak E) integral value adjusted to 100, a simple percentage value of structurally similar peaks in the same region of the spectrum can be calculated. Due to having similar, but slightly different, resonant frequencies peaks A, B, C, D, and F can be presumed to be compounds with a substituted arene moiety similar to that of THC. However, as these peaks have differing chemical shifts than the majority Δ-8 THC product, they are clearly not the same compound. When the peak areas are added together, it is clear that the COA purity values supplied with these products are not consistent with the measured values for each sample.

**Figure 5:**
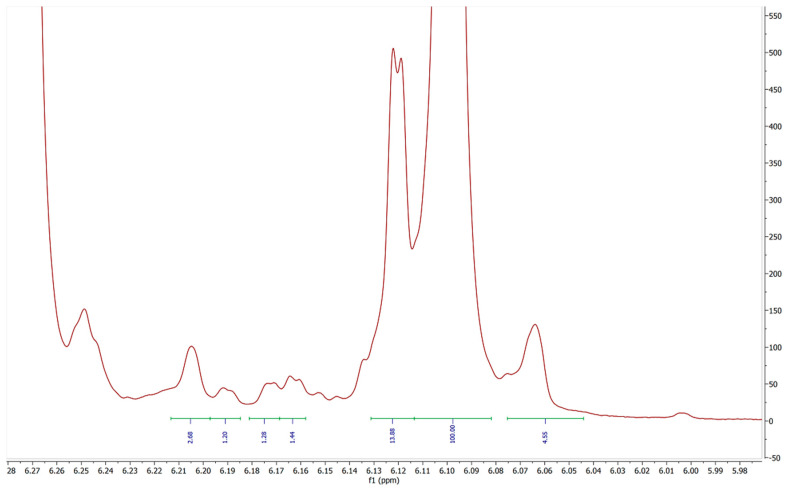
400 MHz ^1^H NMR spectrum of sample 3 from 5.97 to 6.28 ppm.

Due to the general unavailability of 800 MHz class NMR instruments, sample 3 was also analyzed on a 400 MHz NMR for comparison. While integration of the discrete peaks in a 1d proton spectrum is more challenging due to the lower resolution it appears to be a viable option for analysis of these products (Figure 5).

Samples 6 through 10 (Table 1) contain added terpenes with resonances in the region of interest. Therefore, care must be taken when integrating impurities in this region to avoid including terpene signals as impurities. Samples 8, 9, and 10 were supplied with identical COA’s suggesting that they were from the same lot of base product but considering the difference in impurities between sample 8 and the other two this appears to not be the case. Overall, none of the samples analyzed, save for sample 4, are close to having accurate COA purity values even when only investigating a single peak and ignoring the rest of the spectrum. Due to the chemical shift and J-value similarities of this peak and that found on Δ-8 THC, the compounds could reasonably be assumed to contain an arene and thus these products would very likely be UV active and detectable on an HPLC equipped with a UV detector or DAD.

**Figure 6:**
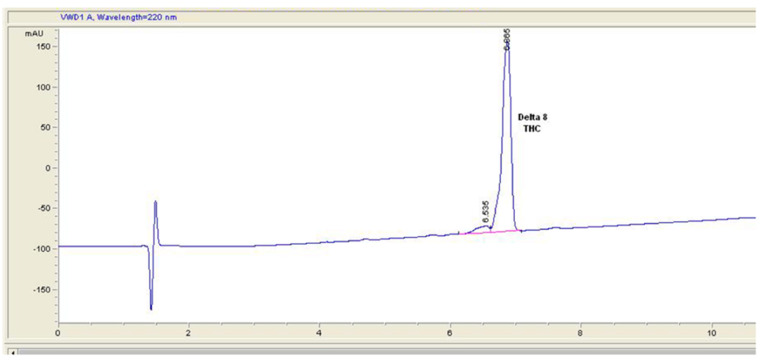
Certificate of Analysis chromatogram of sample 6.

**Figure 7:**
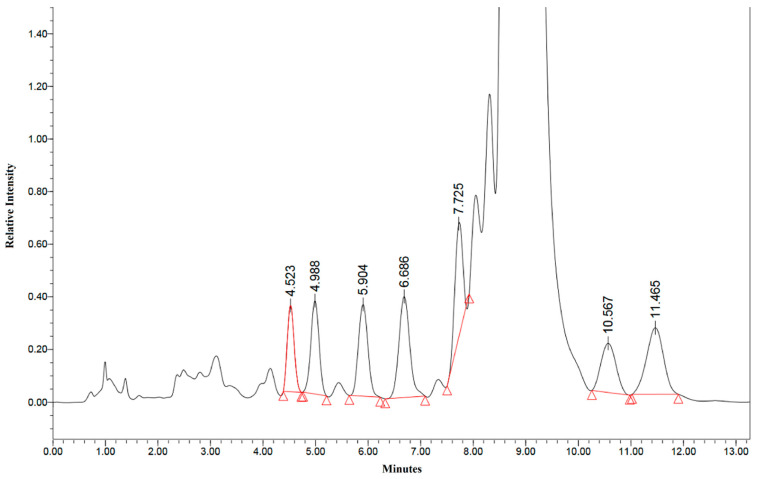
HPLC chromatogram of sample 6 performed during this study. A wavelength of 210 nm was used for the UV detector. The red triangles mark the range of a peak for integration.

Comparing the HPLC chromatogram supplied with sample 4 (Figure 6) to an HPLC-UV performed during this study (Figure 7), it is clear that the HPLC elution method used by the certifying laboratory is inappropriate for detecting impurities in this type of product.

**Figure 8:**
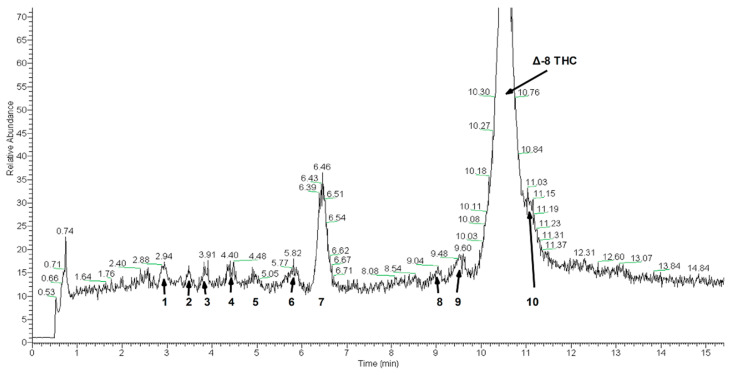
Total ion chromatogram of sample 4 in the positive ion mode and a mass range of m/z = 317 to 750. The peak labels correspond to the peaks listed in Table 3.

Mass spectrometry and MS^2^ analysis of peaks in sample 4 revealed several impurity peaks. Figure 8 is the total ion chromatogram for sample 4 over the mass range of 317–750 Da. Table 2 is a summary of the LC/MS experiment. The peak number in the first table column correspond to the numbers shown in Figure 8. The second column indicates the retention time of the peak. The “major peak” column indicates the mass of the base peak observed for each chromatographic peak. The last column shows the masses of other peaks observed in the mass spectrometry scan.

Table 2 shows the results of several MS/MS experiments. Each of the labeled peaks in Figure 8 were subjected to MS^2^ analysis. The parent ion column in Table 3 is the same as the major peak listed in Table 2. The parent ion was isolated and then fragmented in the MS/MS experiment. The “Base Peak” in Table 3 indicates the mass of the largest ion intensity in the MS/MS spectrum. The last column identifies other fragment ions in the MS/MS spectra.

Analysis of the MS^2^ spectra yielded interesting results showing a variety of what appear to be both impurities from low quality CBD feedstock and known side reaction products from the cyclization reaction used to convert CBD into Δ-8 THC.

Peak 1 exhibits a molecular mass of 328 Da, and as such, this impurity is certainly not CBD or Δ-8 THC. The MS^2^ spectra compares well with published data describing cannabidihexol (CBDH) or tetrahydrocannabihexol. [5] (See supplemental spectrum Peak 1).

Peak 2 also exhibits a molecular mass of 328 Da and possesses a similar MS^2^ spectrum to peak 1. Due to the longer retention time would suggest that this impurity may be an isomer of the impurity in peak 1.

Peak 3 with an odd mass of 253 Da appears to contain an amine. The use of amine containing reagents do not appear in any published literature describing the conversion of CBD to Δ-8 THC and as such the origin of this impurity is unknown. A mass transition of [M-85]^+^ may indicate the presence of a piperidine. However, further work would be required for definitive identification.

Peak 4 exhibits [M-18]^+^, [M-42]^+^, and [M-56]^+^ mass transitions suggesting that it is also a cannabinoid analogue. However, the mass of 344 Da suggests that this compound may be an O-alkylated cannabinoid analogue, which is supported by the [M-46]^+^ mass transition showing the loss of an ethyl ester.

MS^2^ spectra for peak 5 appears to show transitions expected from 5′′-hydroxy-CBD or 5′′-hydroxy-THC. With the characteristic loss of water at [M-18]^+^, an [M-60]^+^ ion suggesting the loss of C_3_H_7_OH, an [M-72]^+^ transition for loss of C_4_H_8_OH, and an [M-74]^+^ transition for a loss of C_4_H_10_OH. Remaining [M + H]^+^ ions of 193 Da and 107 Da are suggestive that this is likely a 5′′-hydroxy CBD or THC analogue. This impurity likely arises from side reactions in the CBD to Δ-8 THC conversion reaction [6].

Peak 6 appears consistent with published MS2 spectra of cannabidivarin (CBDV) [7]. Due to being a metabolite found in cannabis, this is likely an impurity carried over from low purity CBD feedstock.

Peak 7 is quite unusual compared to the other impurities investigated in this study. In MS^2^ spectra the base peak has a mass transition of [M-180]^+^ from the molecular ion suggesting the presence of a hexose moiety in this contaminant. The source for this impurity is unknown and further investigation would be required for full structural elucidation.

Peak 8 with an [M + H]^+^ molecular ion of 315 Da is consistent with MS^2^ spectra of THC or CBD. However, due to the longer retention time of 9 min compared to a retention time of 6.46 min for CBD and the slightly longer retention time for Δ-8 THC of 10.5 min, it would appear to be a CBD or THC isomer.

Peak 9 exhibits MS^2^ spectra similar to peak 8 with a slightly longer retention time of 9.5 min. This is also likely another CBD or THC isomer.

Peak 10 MS^2^ transitions are similar to published spectra of cannabidihexol [6]. However, the [M-42]^+^ base peak, is significantly more intense in spectra collected in this study. This impurity is tentatively identified as cannabidihexol or an isomer thereof.

With several of these impurities now having tentative identifications it appears that the problem with these products is threefold: impure CBD feedstock, poor post-reaction purification, and poor analytical practices during certification of purity. With NMR, HPLC-UV, and HPLC-MS data, it is clear that the current analysis methods need to be improved. Inadequate purification and testing protocols gives rise to a situation where consumers make use of products with far higher levels of impurities than they were led to believe, a situation that could potentially give rise to catastrophic consequences. A less extreme and possibly even more concerning problem arises from considering that failures such as this could lead to a loss of public confidence in laboratory testing of consumer goods altogether.

Even a simple HPLC-UV analysis of these products shows that the certifying labs are failing their customers and consumers by using inappropriate HPLC conditions. While NMR and HPLC-MS could be an arguably more precise methods for detecting and analyzing these impurities the low cost of HPLC is undeniably appealing. HPLC is certainly still a viable method for analysis of these products but certifying laboratories must be vigilant regarding the effectiveness of the methods used in these analyses as demonstrated by the clear failings described above.

## 3. Materials and Methods

### 3.1. Samples

All samples were purchased online or at local retailers. All vaporizer cartridges contained added non-THC hemp extracts in order to convey an organoleptic experience similar to particular strains of cannabis. Most samples contained a certificate of analysis (COA), with some denoting analysis via high performance liquid chromatography (HPLC) equipped with an ultraviolet (UV) or diode array detector (DAD) (Table 4).

### 3.2. H NMR

Approximately 50 mg of each product was dissolved in 600 µL of deuterochloroform (CDCl3 99.8% D, 0.03% *v*/*v* TMS, Acros Organics, Switzerland), and loaded into a 5 mm NMR tube for analysis. The spectrometer used was a Bruker (Rheinstetten, Germany) Avance III spectrometer equipped with a TCI cryoprobe operating at 800.15 MHz. All samples were allowed to thermally equilibrate for 5 min after loading into the magnet. A 16 scan proton experiment (30° pulse, 14 ppm sweep width, 15 s relaxation time based upon 1.4 s T1, 128,000 data points, 300 K sample temperature) was carried out. Spectra was processed using Mestrenova 14.2 (Mestrelab, Santiago de Compostela, Spain).

For comparison, a proton experiment was performed on sample 3 using a Bruker Avance IIIHD spectrometer operating at 400.13 MHz equipped with an inverse probe. All experimental and processing parameters were identical to the 800 MHz experiment.

### 3.3. HPLC Analysis

Approximately 40 mg of sample 4 was dissolved in 1 mL of HPLC grade acetonitrile (Fisher Scientific, Fair Lawn, NJ, USA). HPLC analysis was performed using a Waters (Waters, Milford, MA, USA) 1525 HPLC equipped with a Waters 2487 UV detector operating at 210 nm. An isocratic separation was performed with a 1.4:1 acetonitrile to water mobile phase with a 15 min total run time and flow rate of 1.8 mL per minute. The stationary phase used was a Waters 5 µm C18 4.6 × 150 mm column.

### 3.4. HPLC-Mass Spectrometry

15 mg of sample 4 was dissolved in 1 mL of HPLC grade acetonitrile. Mass spectrometry analysis was performed using a Thermo Finnegan LCQ Deca Plus mass spectrometer equipped with an ESI source and using the positive ion mode. The spray voltage was 5 kV, the sheath gas was set to 70, and the sweep gas was set to 30. Chromatography (Perkin Elmer 200, Waltham, MA, USA) was performed with a 5 µm C18 column with dimensions of 2.1 × 150 mm (GL Sciences, Tokyo, Japan). The mobile phase used was 2.125:1 H_2_O and acetonitrile each with 0.1% *v*/*v* formic acid under isocratic conditions with a flow rate of 0.5 mL per minute. The injection volume was 10 µL. Mass spectra were processed using Xcalibur 2.0.7 (Thermo Scientific, San Jose, CA, USA). MS-MS experiments were performed under identical conditions with a collision energy of 30% using He (UHP grade, Air Products, Allentown, PA, USA) as the collision gas.

## Figures and Tables

**Table 1:** Table of impurity integrals relative to the 5′ proton “E” (Figure 2 and Figure 3). 5′ proton integral calibrated to 100.

Sample	Peak A	Peak B	Peak C	Peak D	Peak E (5′)	Peak F	Total Impurities (% of 5′ peak)	COA Purity Value
1	0.05	4.33	4.86	0.56	100	1.31	11.72	≥99% Δ-8 THC
2	0.16	7.52	6.63	0.24	100	1.47	16.30	94.7% Δ-8 THC
3	0.27	7.5	5.71	0.12	100	1.76	14.76	93.43% Δ-8 THC
4	0.31	2.9	12.74	0.47	100	0.4	17.29	87.1% Δ-8 THC
5	0	2.92	12.87	0.31	100	0.48	14.50	93.4% Δ-8 THC
6	0.09	4.44	6.05	4.49	100	0.83	14.54	92.96% Δ-8 THC
7	0.44	3.29	5.1	0	100	0.22	8.28	No COA supplied
8	0.26	2.64	5.35	0.12	100	2.48	10.04	93.44% Δ-8 THC
9	0.08	3.63	6.28	0.05	100	2.11	11.30	93.44% Δ-8 THC
10	0.09	4.33	6.65	0	100	0.47	11.11	93.44% Δ-8 THC

**Table 2:** Summary of peaks observed in positive mode total ion chromatogram.

Peak	Retention Time, Min	Major Peak [M + H], Da	Minor Peaks (>60% Intensity Relative to Base Peak), Da
1	2.94	329.22	270.44, 254.42, 212.84, 166.84
2	3.65	329.13	315.20, 262.60, 212.79, 207.74, 166.86
3	3.91	254.04	330.23, 329.11, 254.60, 212.79, 207.67, 166.86
4	4.40	345.11	311.18, 246.48, 166.86
5	5.05	331.28	166.87
6	5.82	287.29	None
7	6.46	331.13	None
8	9.04	315.12	None
9	9.60	315.24	None
10	11.03	329.19	315.20

**Table 3:** Summary of peaks observed in MS^2^ spectra.

Peak	T_R_ (min)	Parent Ion [M + H], Da	Base Peak, Da	Fragment Ions, Da
1	2.94	329.22	287.15	311.10, 301.30, 287.15, 272.97, 271.07, 245.17
2	3.65	329.13	287.07	311.08, 301.04, 287.07, 273.01, 271.14, 269.11, 259.10, 245.14, 231.09, 217.11
3	3.91	254.04	196.95	238.89, 235.81, 218.19, 217.15, 208.88, 196.95, 196.14, 194.80, 168.53, 160.90
4	4.40	345.11	327.02	327.02, 317.09, 303.02, 298.99, 289.87
5	5.05	331.28	313.12	313.12, 289.00, 273.11, 271.04, 259.01, 193.12, 106.92
6	5.82	287.29	231.07	269.09, 245.07, 231.07, 207.07, 205.06, 193.01, 165.07, 135.03
7	6.46	331.13	150.87	313.04, 289.09, 243.13, 233.05, 150.87, 107.04
8	9.04	315.12	259.07	297.16, 273.05, 259.07, 245.11, 233.06, 221.01, 207.05, 193.08, 181.04, 134.97
9	9.60	315.24	259.08	297.12, 273.06, 259.08, 235.08, 233.09, 231.15, 193.10, 181.04, 135.01
10	11.03	329.19	287.07	311.12, 287.07, 286.10, 273.04
Δ-8 THC	10.50	315	259	297, 235, 233, 193, 135
CBD	5.13	315	259	297, 235, 233, 193, 135

**Table 4:** List of samples studied.

Sample	Type of Sample	Color of Sample	Terpenes Added?	Certificate of Analysis (COA)
1	Distillate	Clear	N/A	≥99% Δ-8 THC
2	Distillate	Clear	N/A	94.7% Δ-8 THC
3	Distillate	Pink-Brown	N/A	93.43% Δ-8 THC
4	Distillate	Brown	N/A	87.1% Δ-8 THC
5	Distillate	Light Yellow	N/A	93.4% Δ-8 THC
6	Vaporizer Cartridge	Yellow-Brown	Yes	92.96% Δ-8 THC
7	Vaporizer Cartridge	Yellow-Brown	Yes	No COA Supplied
8	Vaporizer Cartridge	Yellow	Yes	93.44% Δ-8 THC
9	Vaporizer Cartridge	Yellow	Yes	93.44% Δ-8 THC
10	Vaporizer Cartridge	Yellow	Yes	93.44% Δ-8 THC

## Data Availability

All data are contained within the article and supplemental information. The original data presented in this study are available on request.

## Supplementary Materials

The following supporting information can be downloaded at: https://www.mdpi.com/article/10.3390/molecules27206924/s1, The supporting information contains the 800 MHz ^1^H NMR spectra for each sample listed in Table 1 and MS/MS spectra of each sample listed in Table 4 of the manuscript.

## Sample Availability

Samples of the compounds are available from the authors.
