# Supplementary material for: Delta-8 Tetrahydrocannabinol Product Impurities
Source: Molecules. 2022 Oct 15;27(20):6924. doi: 10.3390/molecules27206924 (PMC9608670; doi:10.3390/molecules27206924)
Supplement: Supplementary file 1 [file molecules-27-06924-s001.zip › molecules-1958426-supplementary.pdf]

# Delta-8 Tetrahydrocannabinol Product Impurities

Colleen L. Ray [1], Madison P. Bylo [1], Jonny Pesacaglia [1], James A. Gawenis [2] and C. Michael Greenlief [1,*]

[1] Department of Chemistry, University of Missouri, 601 S. College Avenue, Columbia, MO 65211, USA

[2] Sweetwater Science Laboratories, Glasgow, MO 65264, USA

* Corresponding Author: Email address: greenliefm@missouri.edu (C.M. Greenlief)

Contents

800 MHz $^{1}$H NMR spectra of each sample listed in Table 1 of the manuscript.
MS/MS spectra of each sample listed in Table 4 of the manuscript

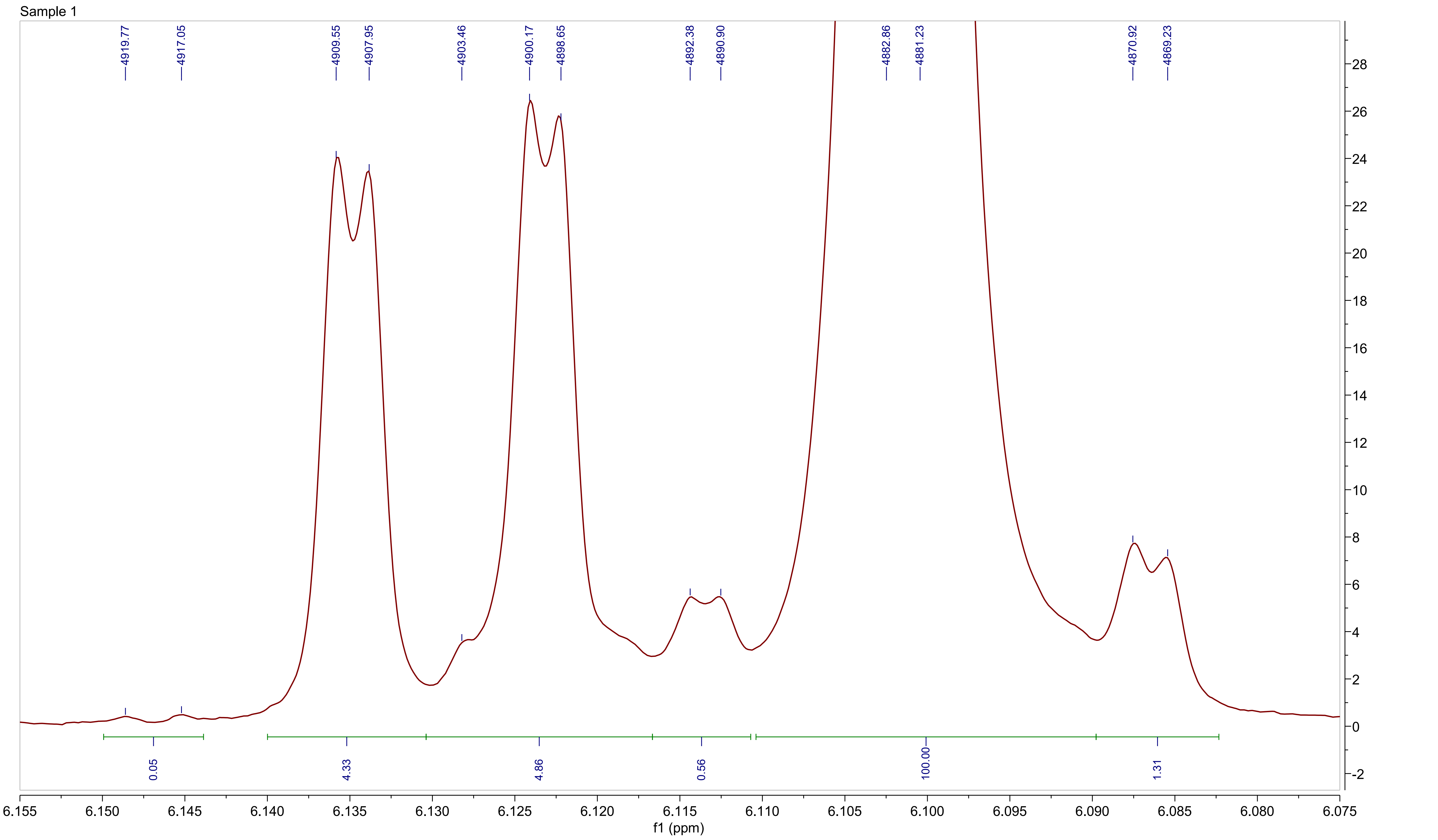
Sample 1
4919.77
4917.05
4909.55
4907.95
4903.46
4900.17
4898.65
4892.38
4890.90
4882.86
4881.23
4870.92
4869.23
0.05
4.33
4.86
0.56
100.00
1.31
f1 (ppm)

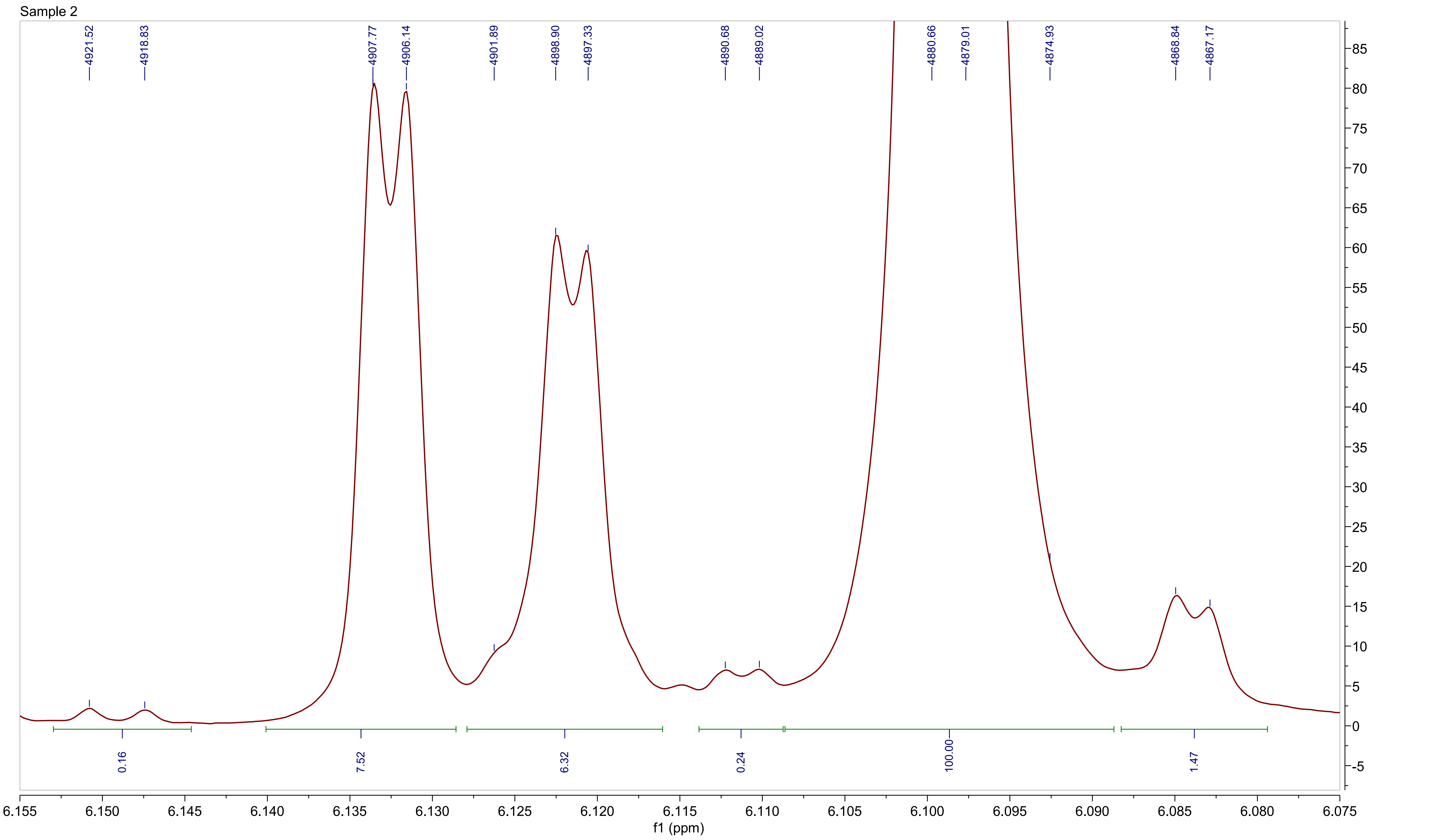

Sample 2
4921.52
4918.83
4907.77
4906.14
4901.89
4898.90
4897.33
4890.68
4889.02
4880.66
4879.01
4874.93
4868.84
4867.17
0.16
7.52
6.32
0.24
100.00
1.47
f1 (ppm)

Sample 3

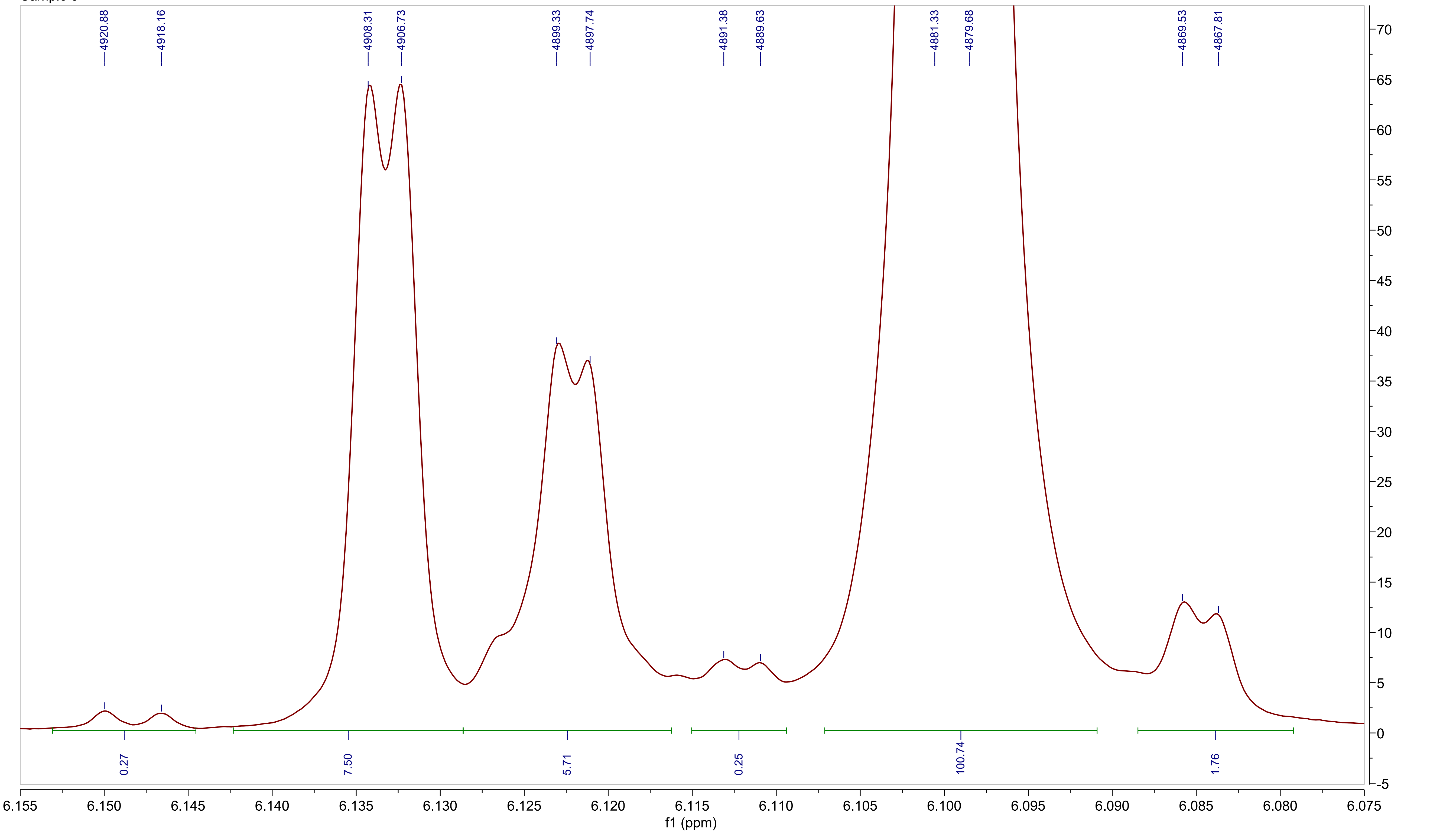

4920.88
4918.16
4908.31
4906.73
4899.33
4897.74
4891.38
4889.63
4881.33
4879.68
4869.53
4867.81
0.27
7.50
5.71
0.25
100.74
1.76
f1 (ppm)

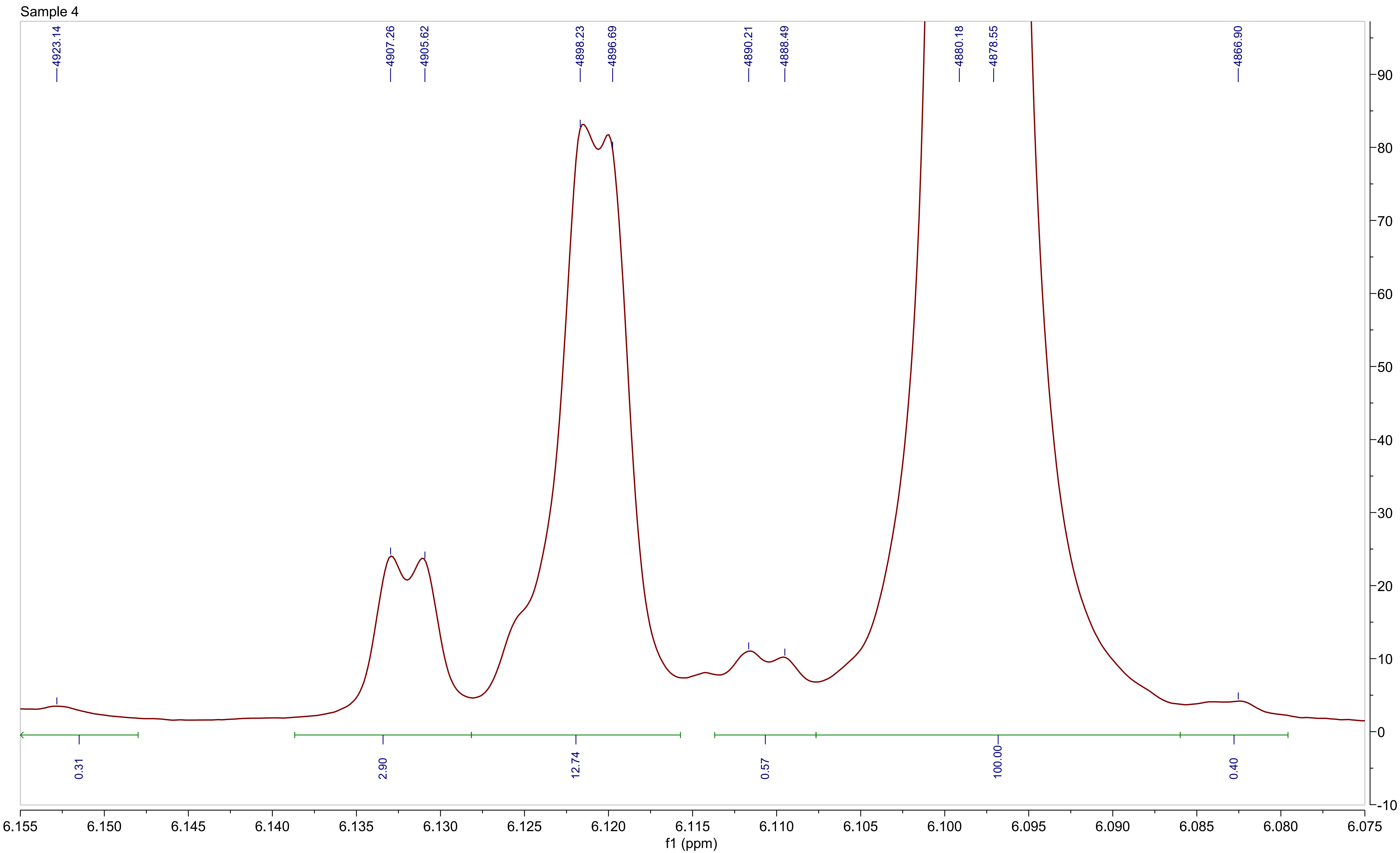

Sample 4
4923.14
4907.26
4905.62
4898.23
4896.69
4890.21
4888.49
4880.18
4878.55
4866.90
0.31
2.90
12.74
0.57
100.00
0.40
90
80
70
60
50
40
30
20
10
0
-10
6.155
6.150
6.145
6.140
6.135
6.130
6.125
6.120
6.115
6.110
6.105
6.100
6.095
6.090
6.085
6.080
6.075
f1 (ppm)

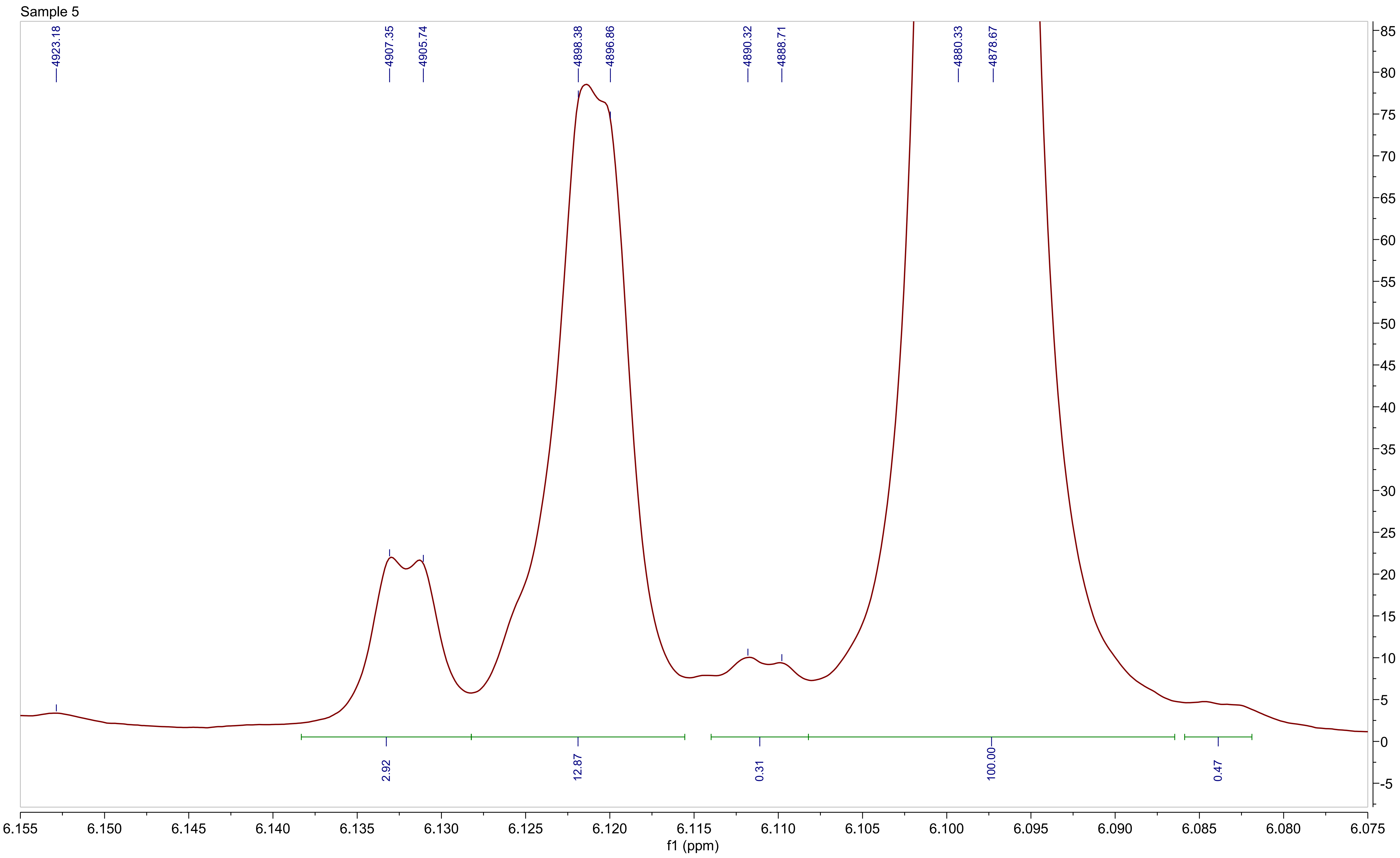
Sample 5
4923.18
4907.35
4905.74
4898.38
4896.86
4890.32
4888.71
4880.33
4878.67
2.92
12.87
0.31
100.00
0.47
f1 (ppm)

Sample 6

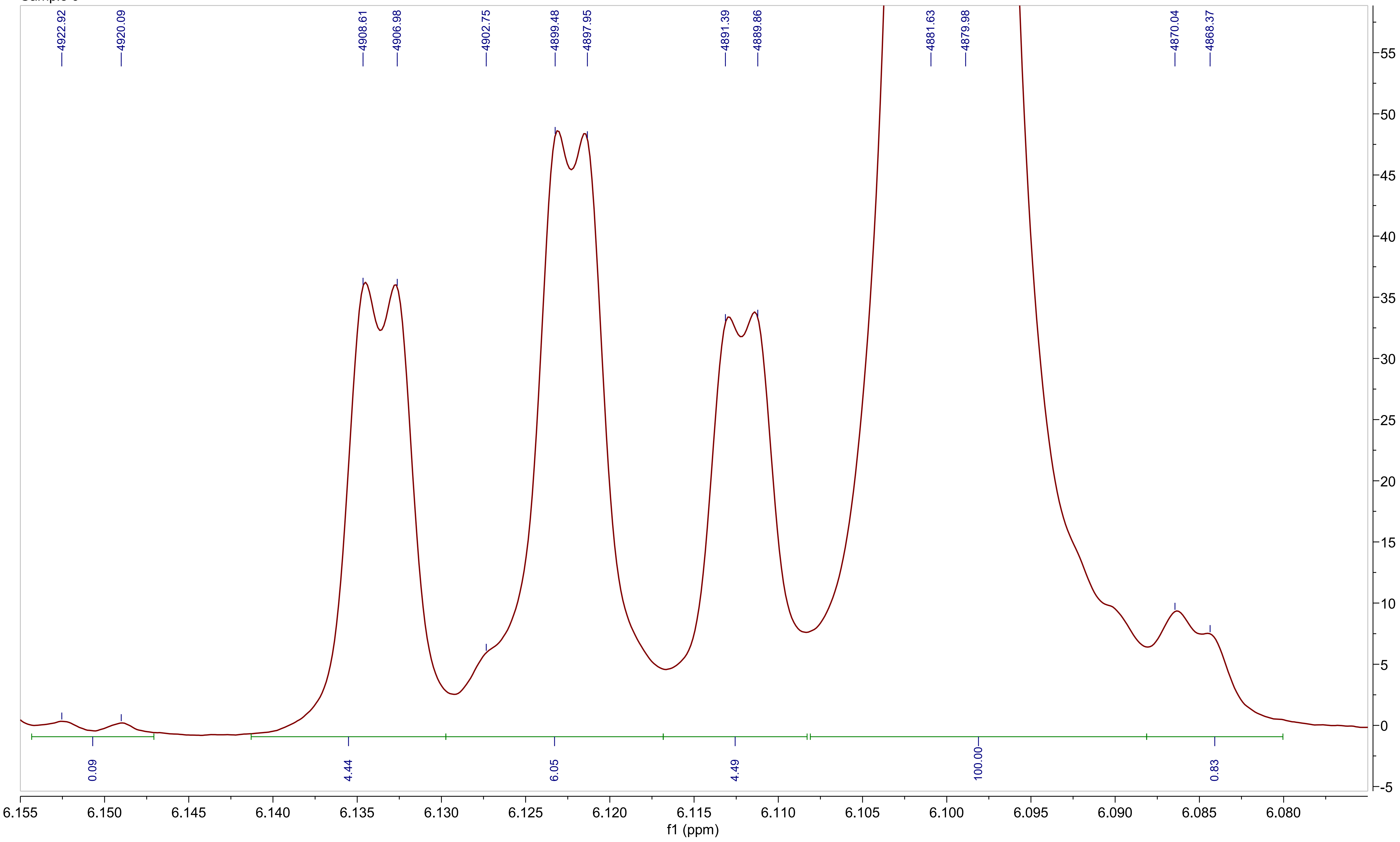

4922.92
4920.09
4908.61
4906.98
4902.75
4899.48
4897.95
4891.39
4889.86
4881.63
4879.98
4870.04
4868.37
0.09
4.44
6.05
4.49
100.00
0.83
f1 (ppm)

Sample 7

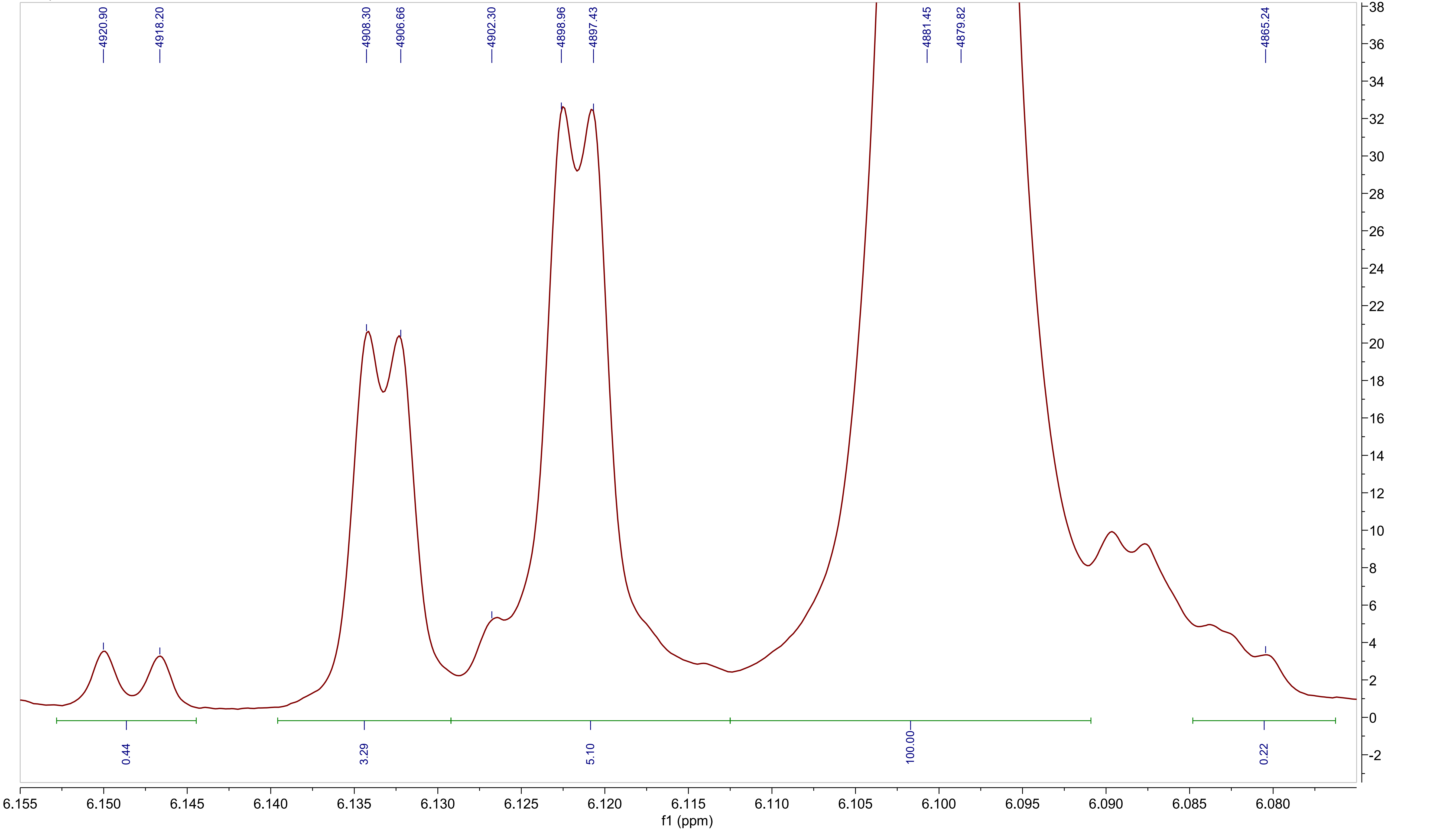

4920.90
4918.20
4908.30
4906.66
4902.30
4898.96
4897.43
4881.45
4879.82
4865.24
0.44
3.29
5.10
100.00
0.22
6.155
6.150
6.145
6.140
6.135
6.130
6.125
6.120
6.115
6.110
6.105
6.100
6.095
6.090
6.085
6.080
f1 (ppm)
38
36
34
32
30
28
26
24
22
20
18
16
14
12
10
8
6
4
2
0
-2

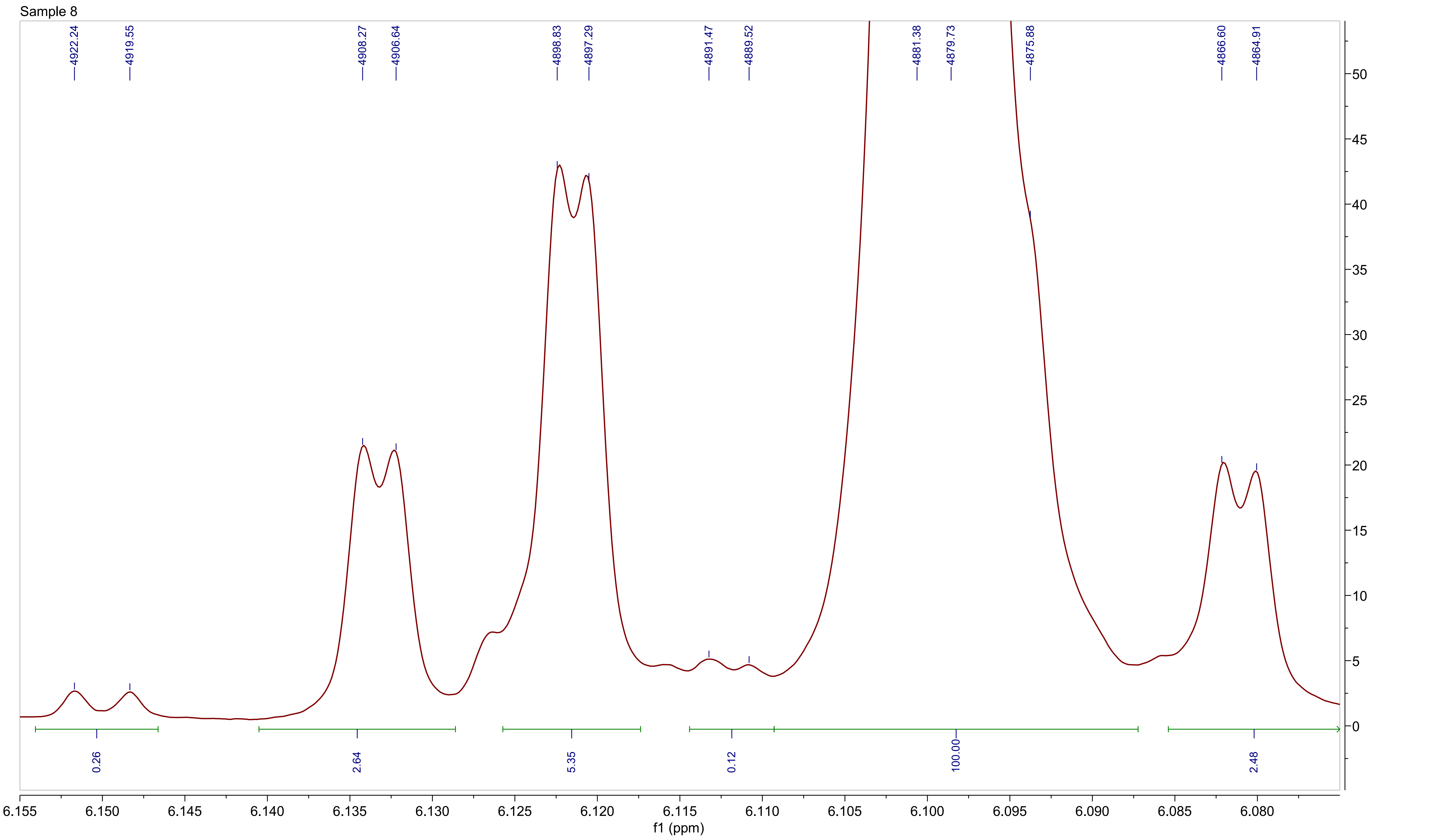
Sample 8
4922.24
4919.55
4908.27
4906.64
4898.83
4897.29
4891.47
4889.52
4881.38
4879.73
4875.88
4866.60
4864.91
0.26
2.64
5.35
0.12
100.00
2.48
f1 (ppm)

Sample 9

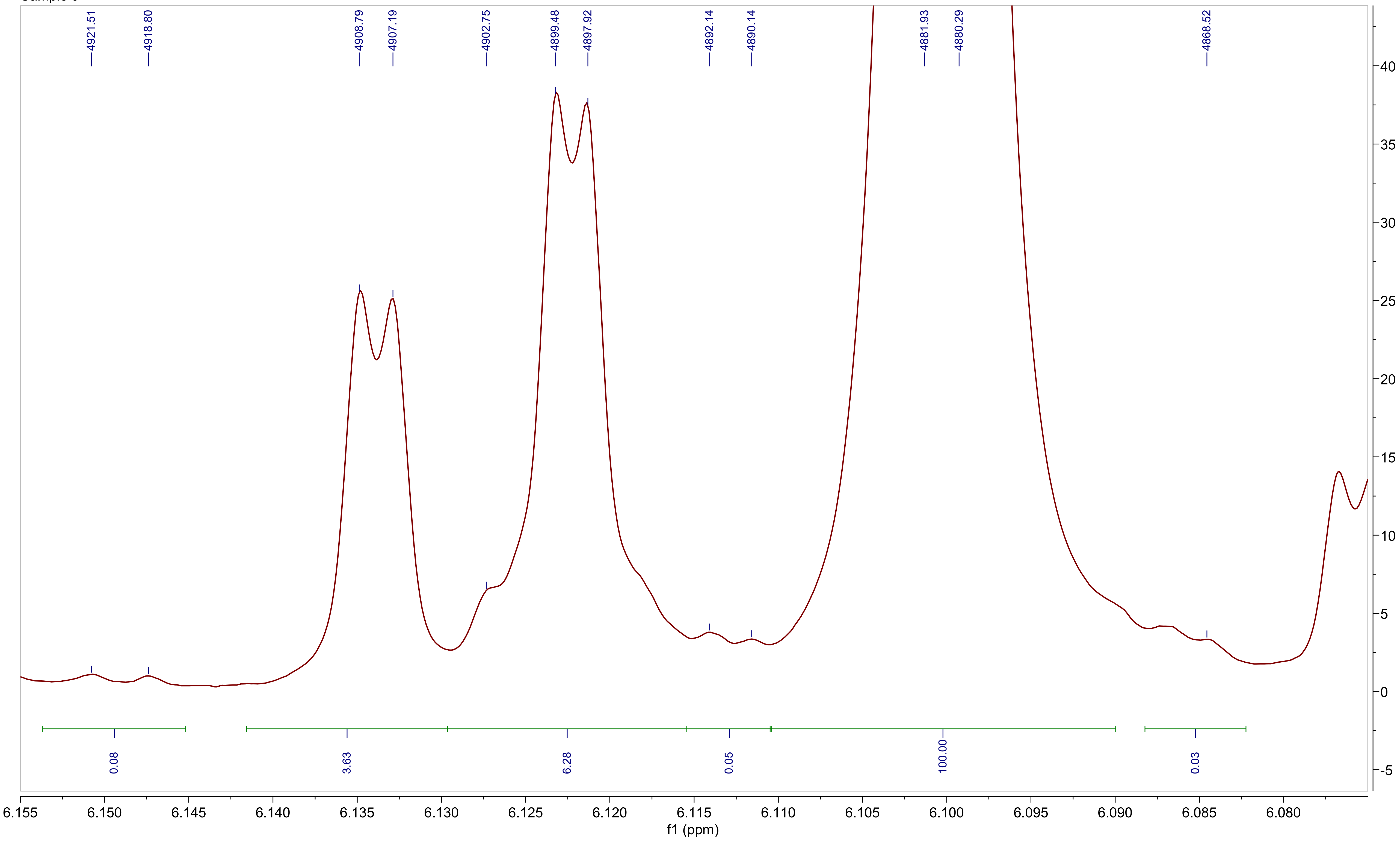
4921.51
4918.80
4908.79
4907.19
4902.75
4899.48
4897.92
4892.14
4890.14
4881.93
4880.29
4868.52
0.08
3.63
6.28
0.05
100.00
0.03
f1 (ppm)

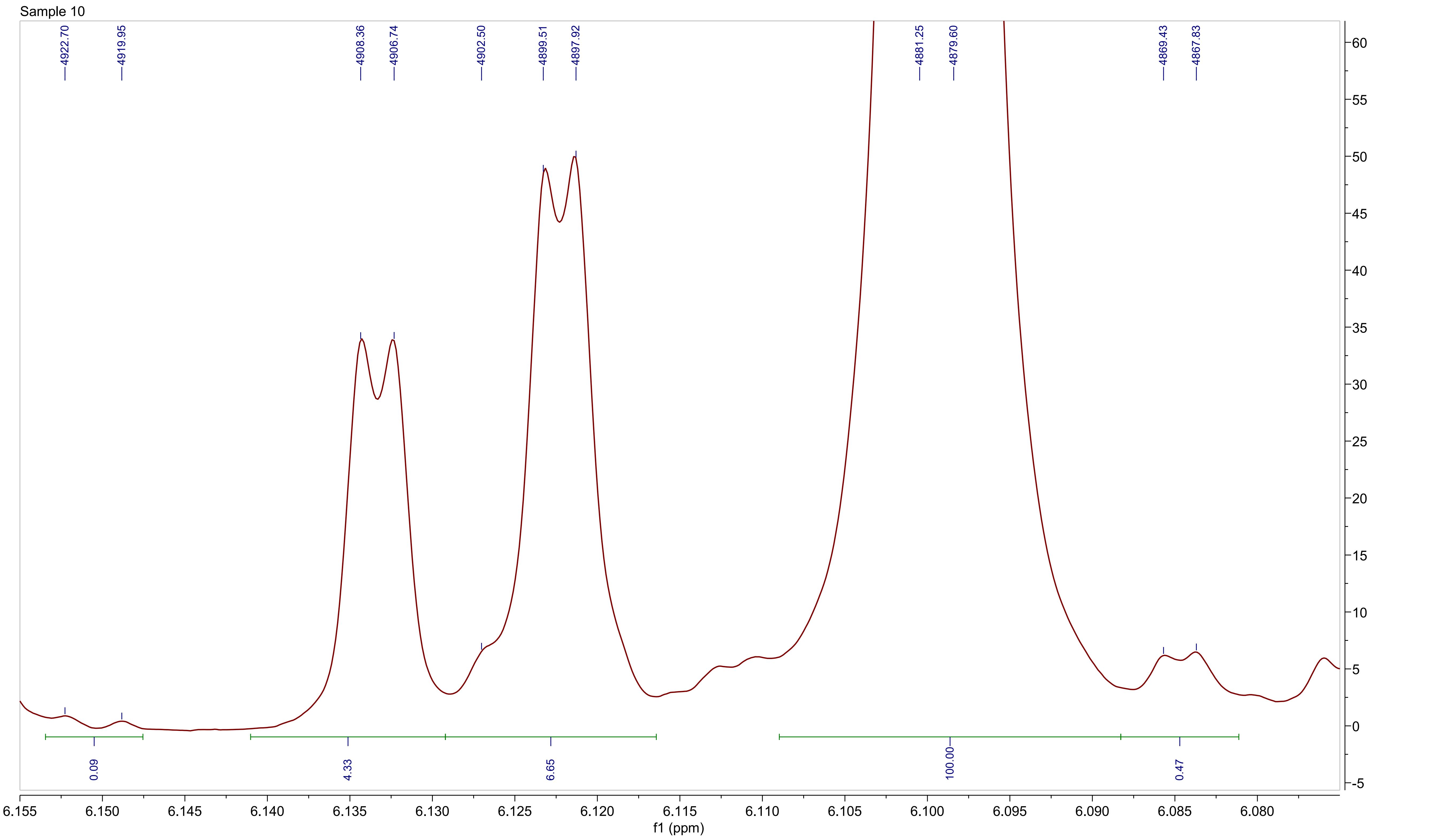

Sample 10
4922.70
4919.95
4908.36
4906.74
4902.50
4899.51
4897.92
4881.25
4879.60
4869.43
4867.83
0.09
4.33
6.65
100.00
0.47
6.155
6.150
6.145
6.140
6.135
6.130
6.125
6.120
6.115
6.110
6.105
6.100
6.095
6.090
6.085
6.080
f1 (ppm)
60
55
50
45
40
35
30
25
20
15
10
5
0
-5

Peak 1

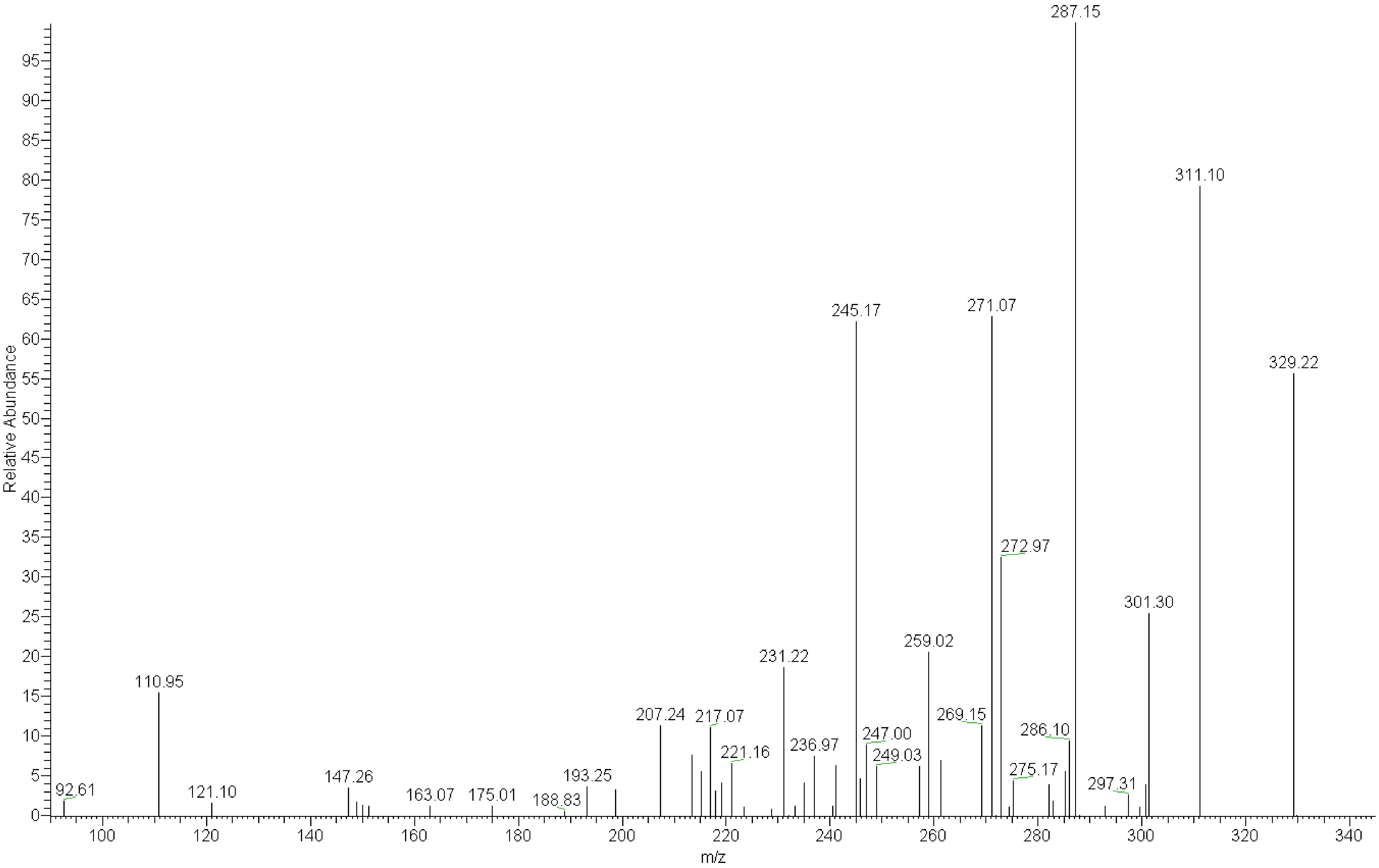

Relative Abundance
m/z
287.15
311.10
329.22
245.17
271.07
272.97
301.30
259.02
231.22
110.95
207.24
217.07
221.16
236.97
247.00
249.03
269.15
286.10
275.17
297.31
147.26
163.07
175.01
188.83
193.25
121.10
92.61

Peak 2

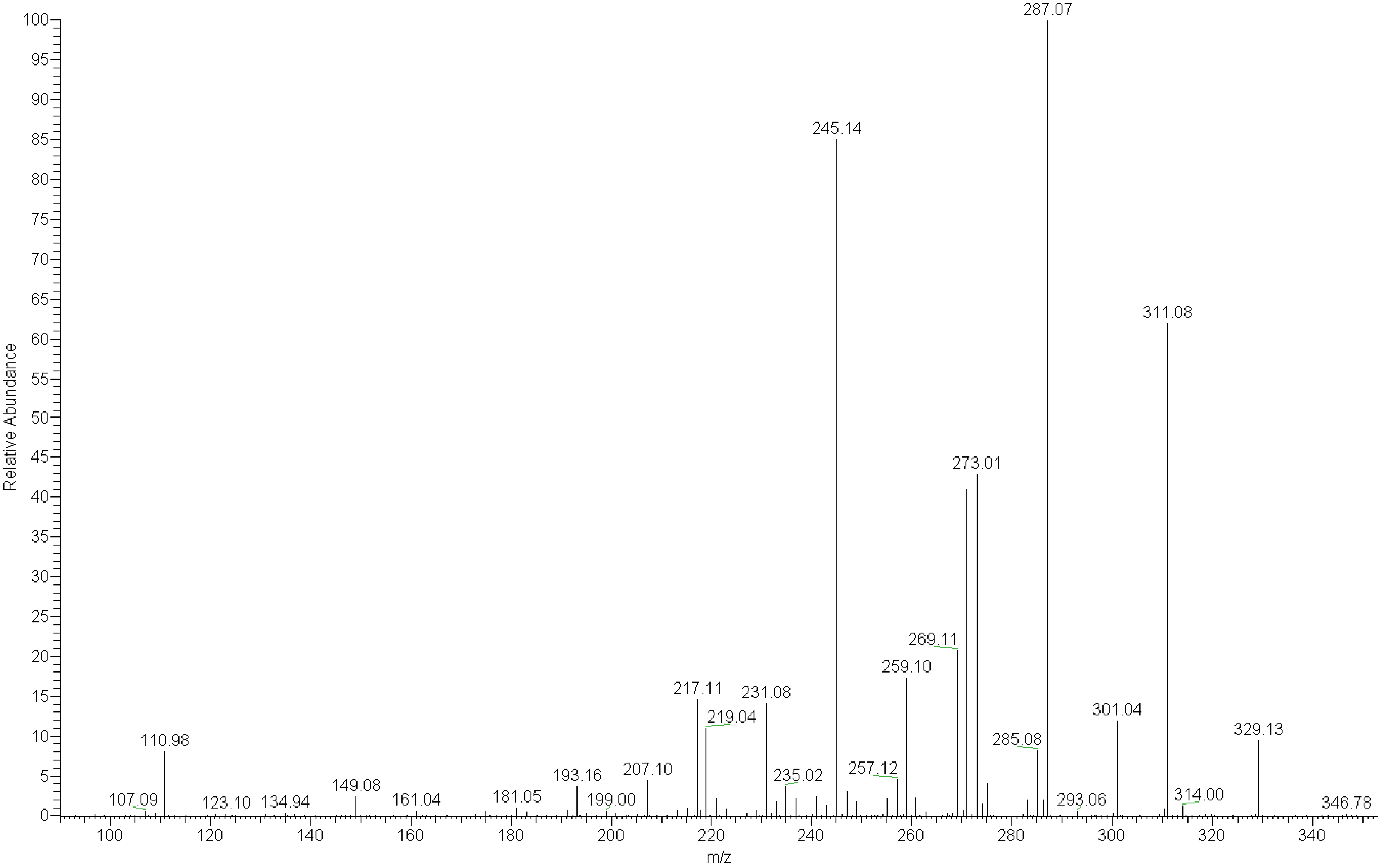

287.07
245.14
311.08
273.01
269.11
259.10
217.11
231.08
219.04
301.04
329.13
285.08
110.98
207.10
193.16
257.12
235.02
149.08
314.00
181.05
107.09
161.04
199.00
293.06
123.10
134.94
346.78
Relative Abundance
m/z

Peak 3

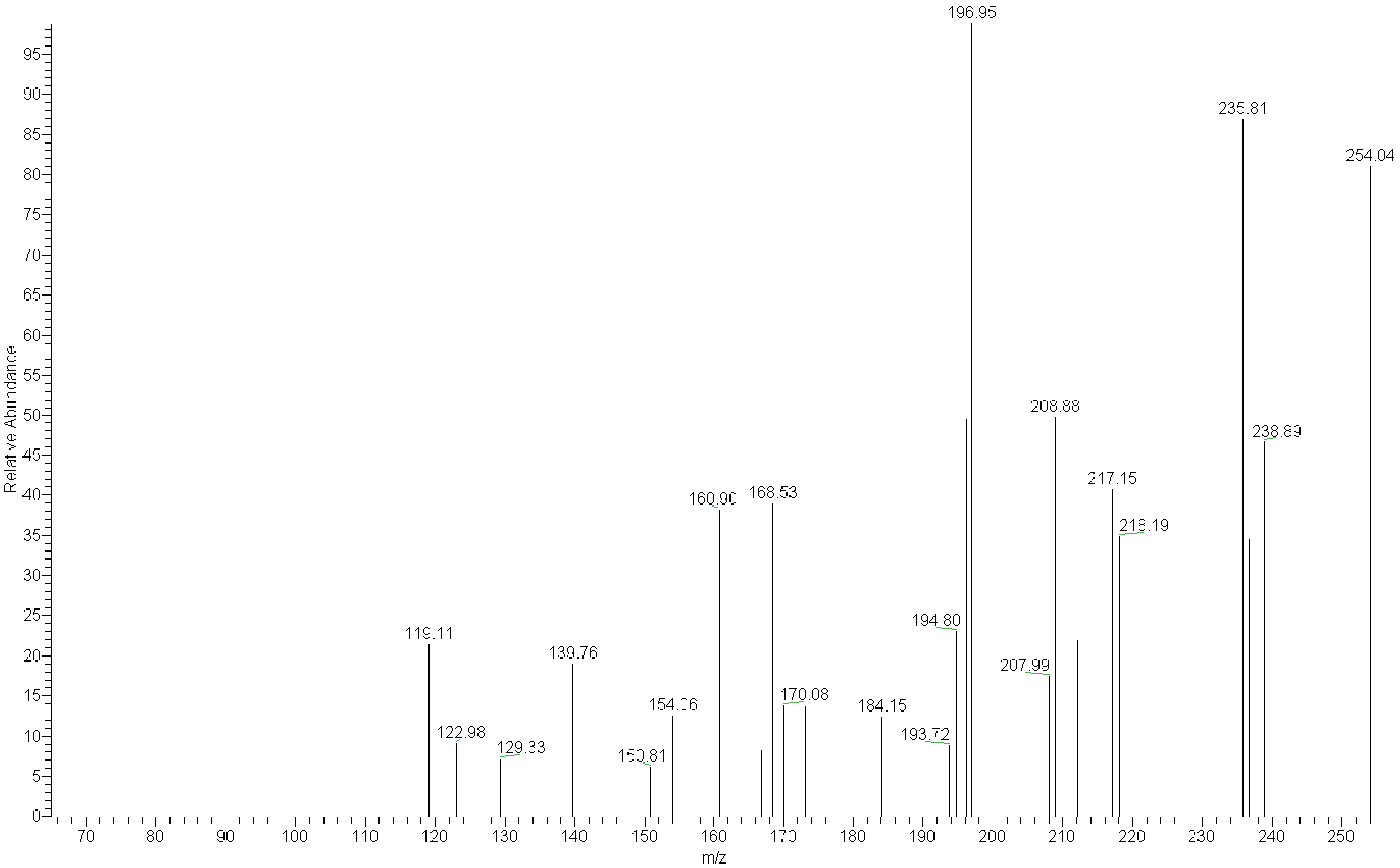
196.95
235.81
254.04
208.88
238.89
217.15
218.19
160.90
168.53
194.80
119.11
139.76
207.99
170.08
154.06
184.15
122.98
129.33
193.72
150.81
Relative Abundance
m/z

Peak 4

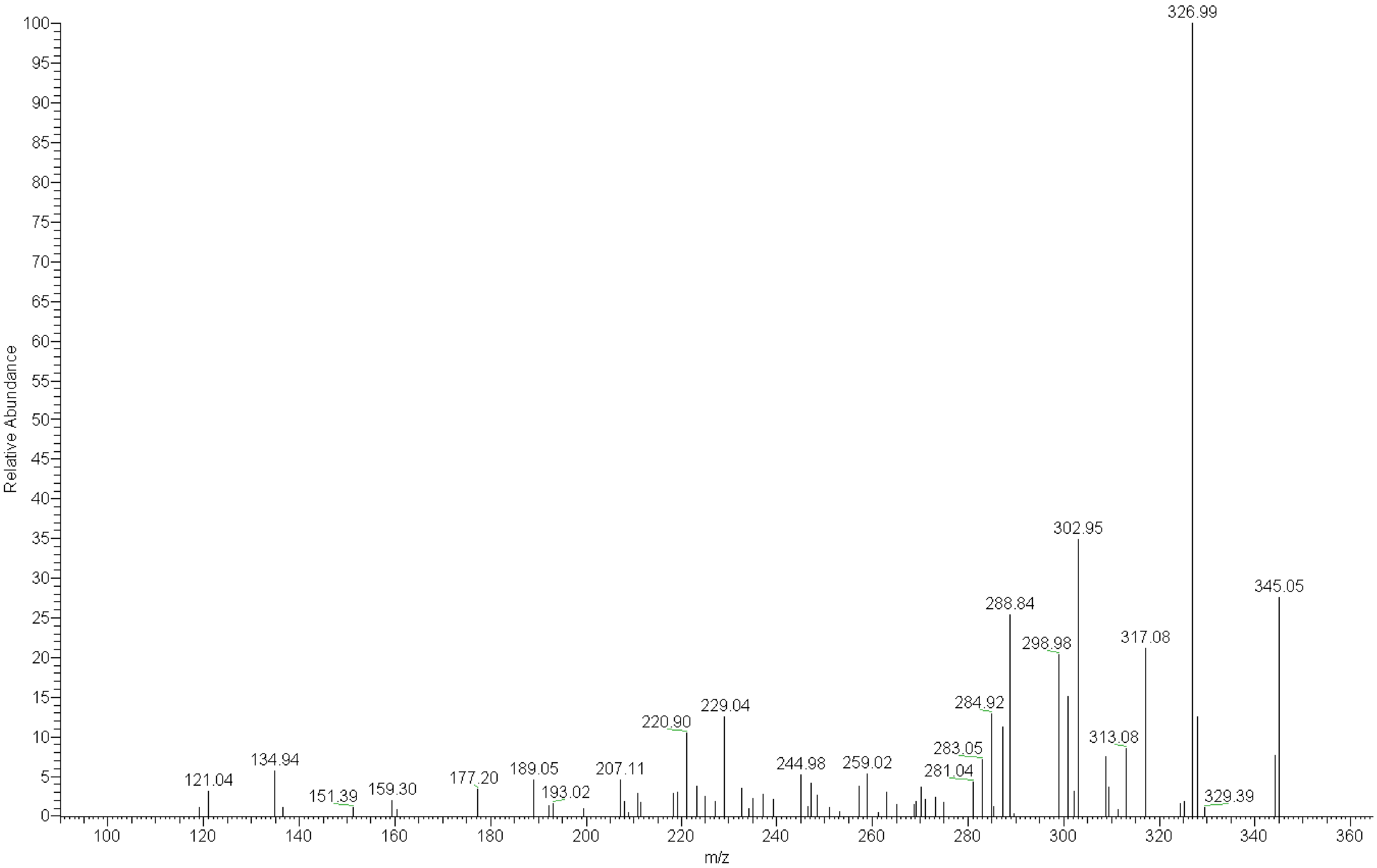

326.99
302.95
345.05
288.84
298.98
317.08
284.92
229.04
220.90
313.08
283.05
134.94
244.98
259.02
281.04
121.04
151.39
159.30
177.20
189.05
193.02
207.11
329.39
Relative Abundance
m/z

Peak 5

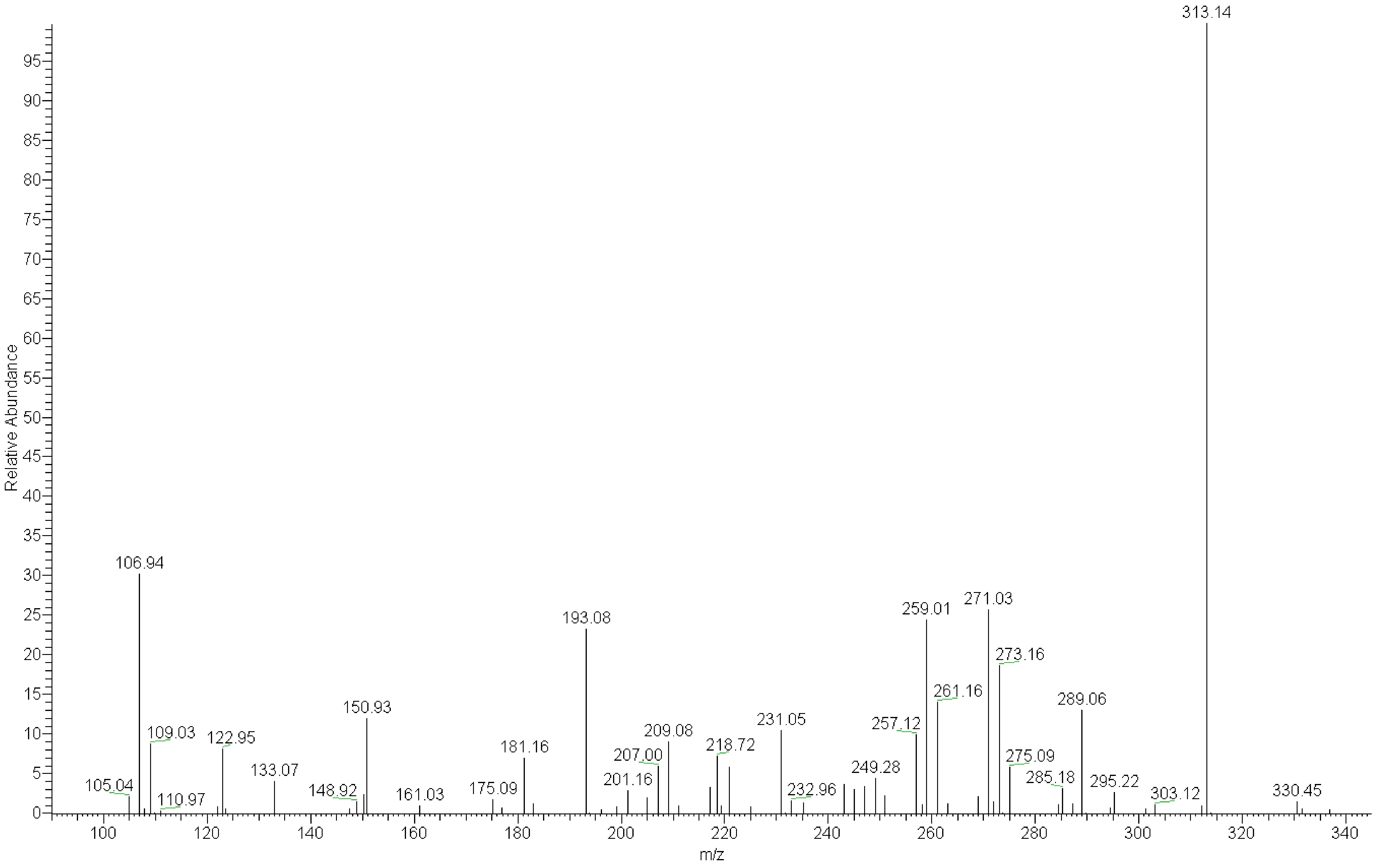

313.14
106.94
193.08
259.01
271.03
273.16
261.16
289.06
150.93
231.05
257.12
209.08
109.03
122.95
218.72
181.16
207.00
275.09
249.28
133.07
201.16
285.18
105.04
295.22
148.92
232.96
175.09
303.12
330.45
161.03
110.97
Relative Abundance
m/z

Peak 6

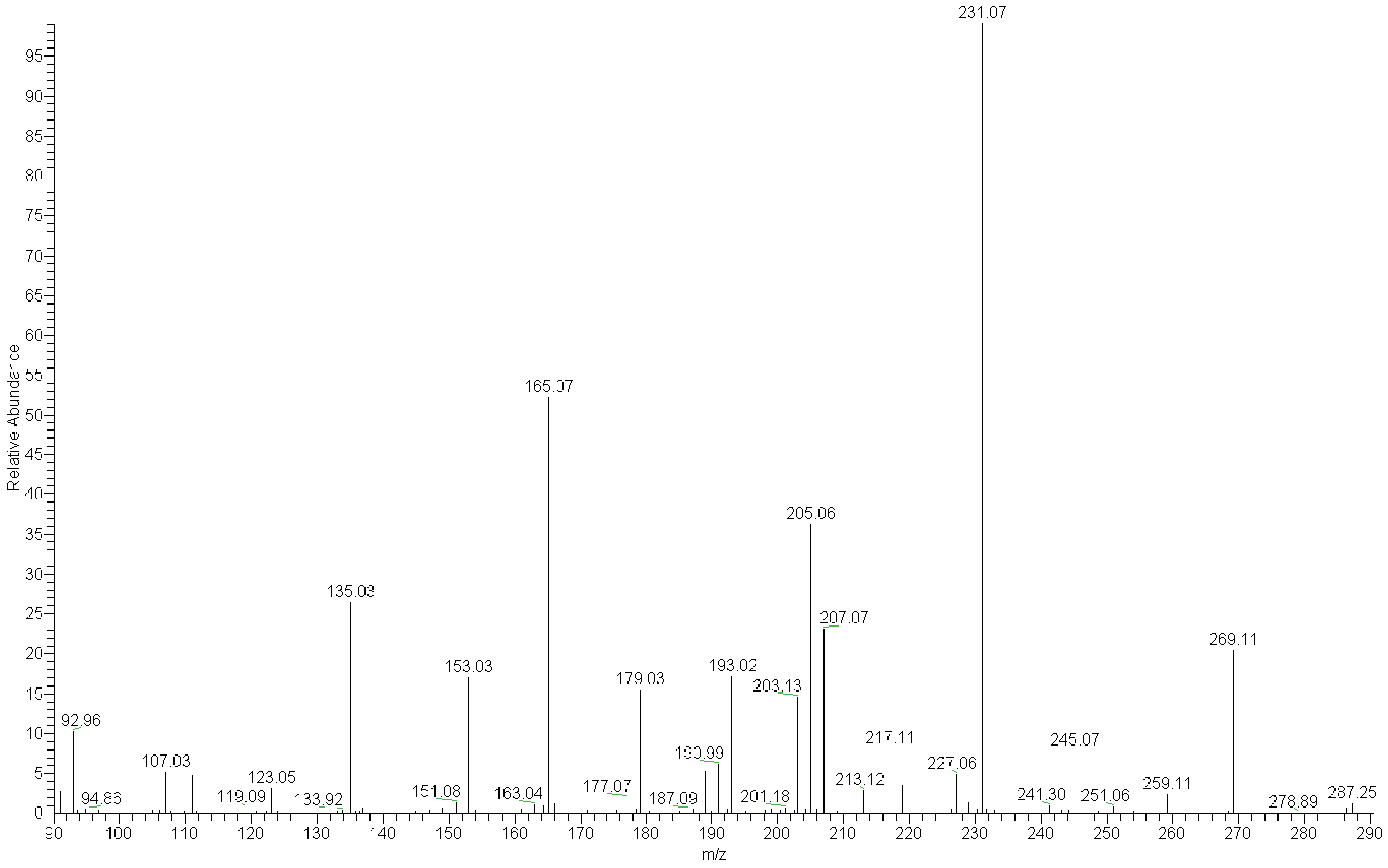

231.07
165.07
205.06
135.03
207.07
269.11
153.03
179.03
193.02
203.13
92.96
217.11
245.07
190.99
107.03
227.06
123.05
213.12
259.11
151.08
163.04
177.07
241.30
251.06
287.25
94.86
119.09
133.92
187.09
201.18
278.89
Relative Abundance
m/z

Peak 7

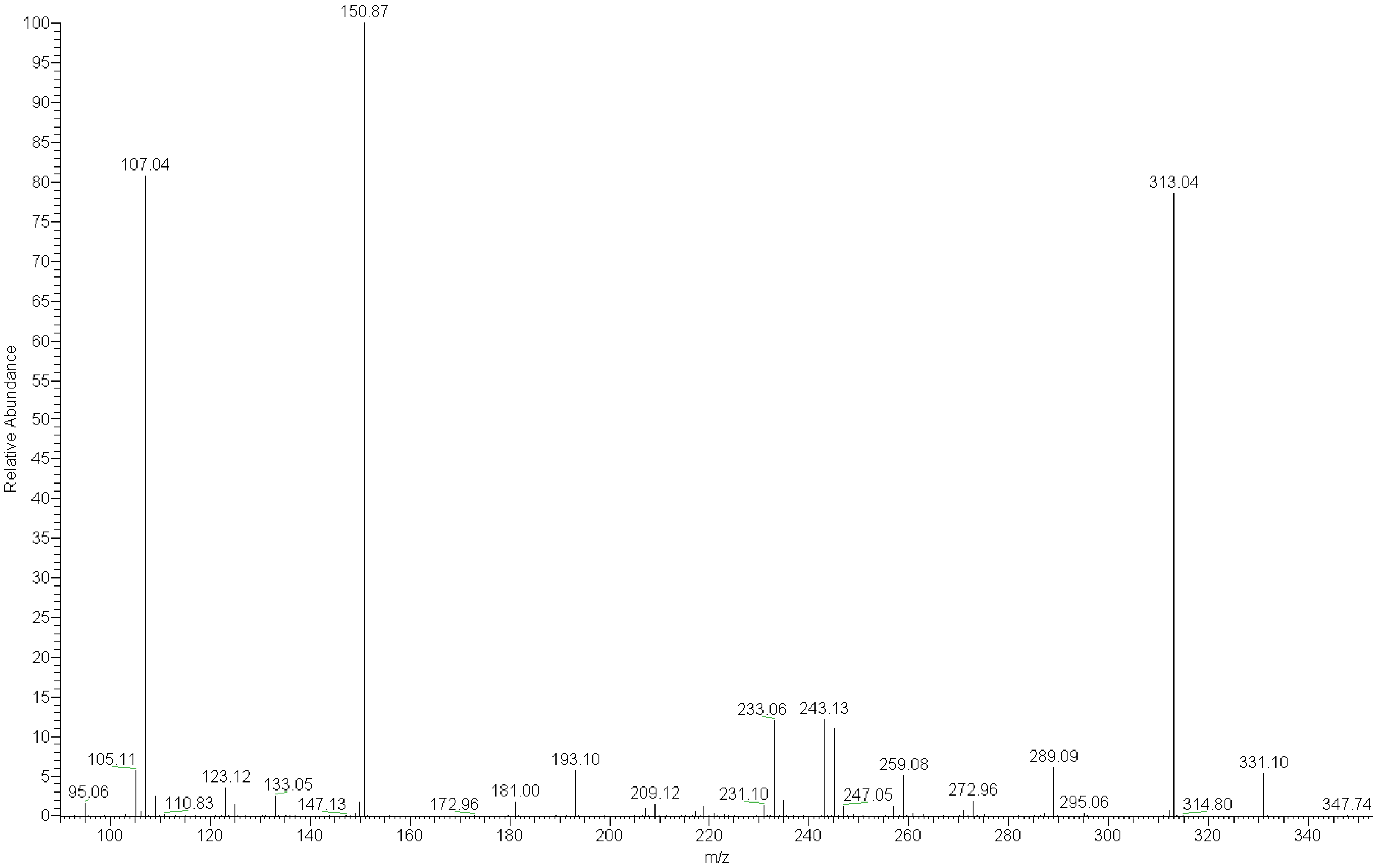

Relative Abundance
m/z
150.87
107.04
313.04
105.11
95.06
110.83
123.12
133.05
147.13
172.96
181.00
193.10
209.12
231.10
233.06
243.13
247.05
259.08
272.96
289.09
295.06
314.80
331.10
347.74

Peak 8

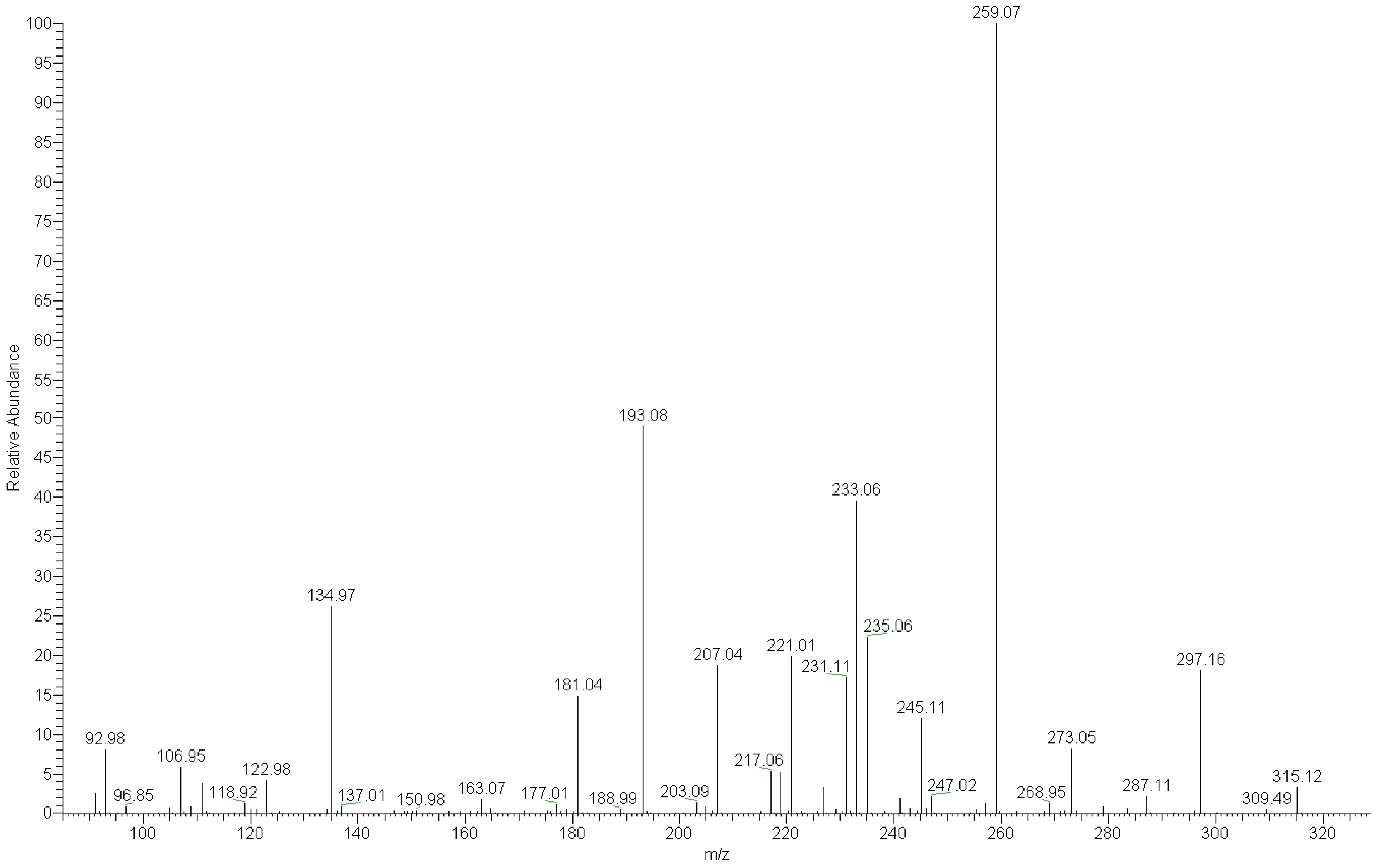

Relative Abundance
m/z
259.07
193.08
233.06
134.97
235.06
221.01
207.04
231.11
297.16
181.04
245.11
273.05
92.98
106.95
122.98
217.06
315.12
96.85
118.92
137.01
150.98
163.07
177.01
188.99
203.09
247.02
268.95
287.11
309.49

Peak 9

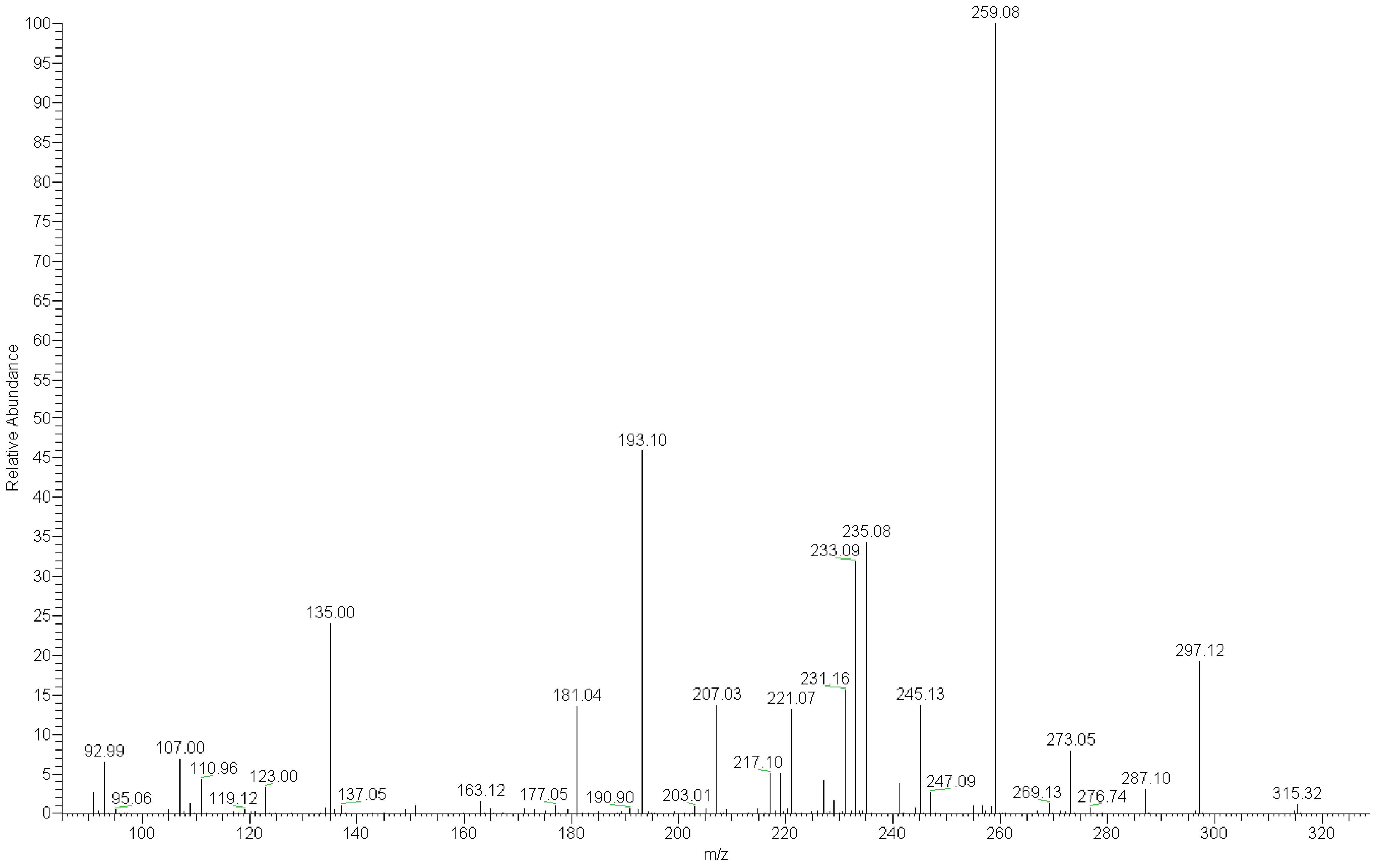

Relative Abundance
m/z
259.08
193.10
235.08
233.09
135.00
297.12
231.16
181.04
207.03
245.13
221.07
273.05
92.99
107.00
110.96
123.00
217.10
287.10
247.09
95.06
119.12
137.05
163.12
177.05
190.90
203.01
269.13
276.74
315.32

Peak 10

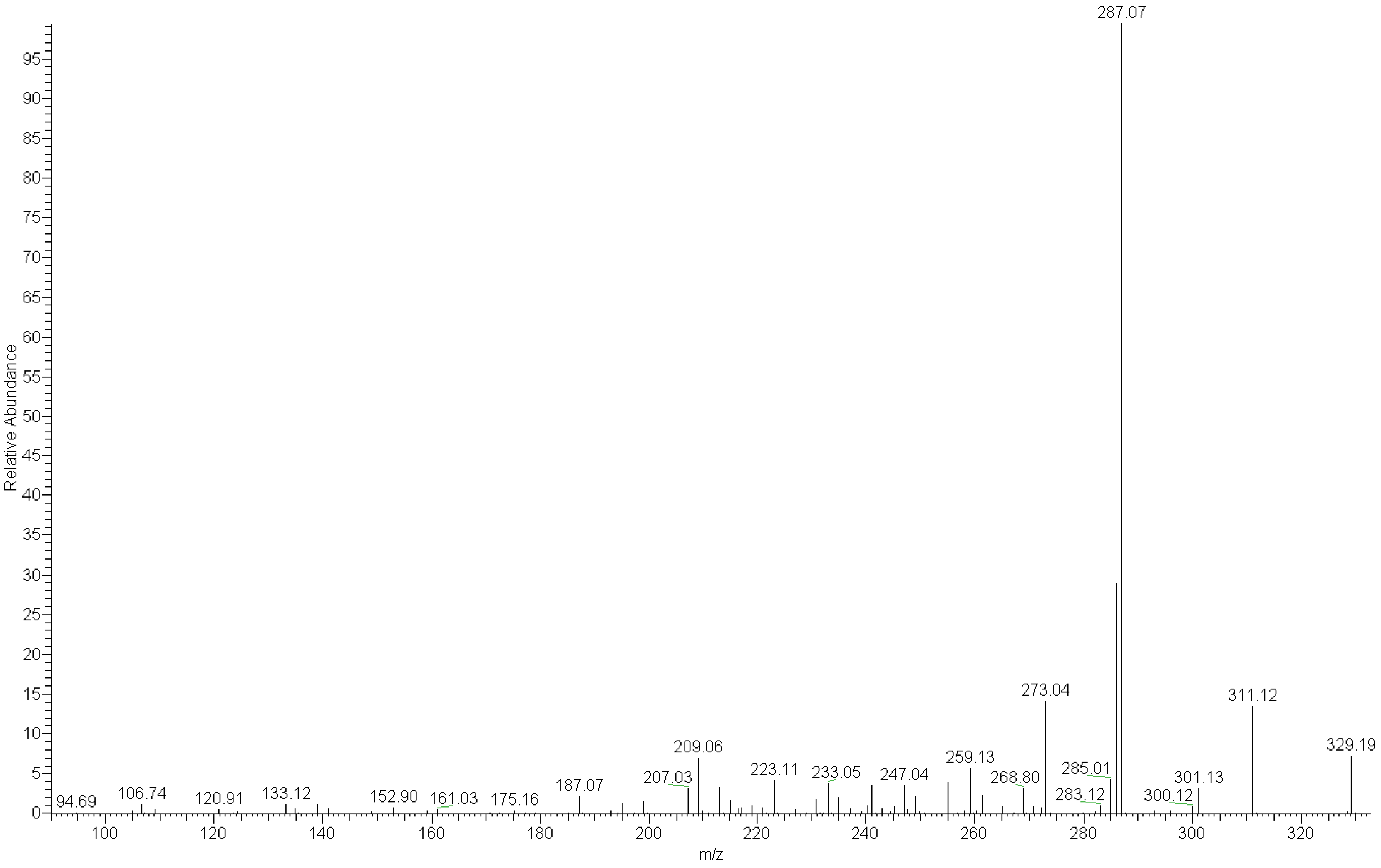

287.07
273.04
311.12
329.19
209.06
223.11
233.05
247.04
259.13
268.80
285.01
283.12
301.13
300.12
207.03
187.07
175.16
161.03
152.90
133.12
120.91
106.74
94.69
Relative Abundance
m/z

Δ- THC

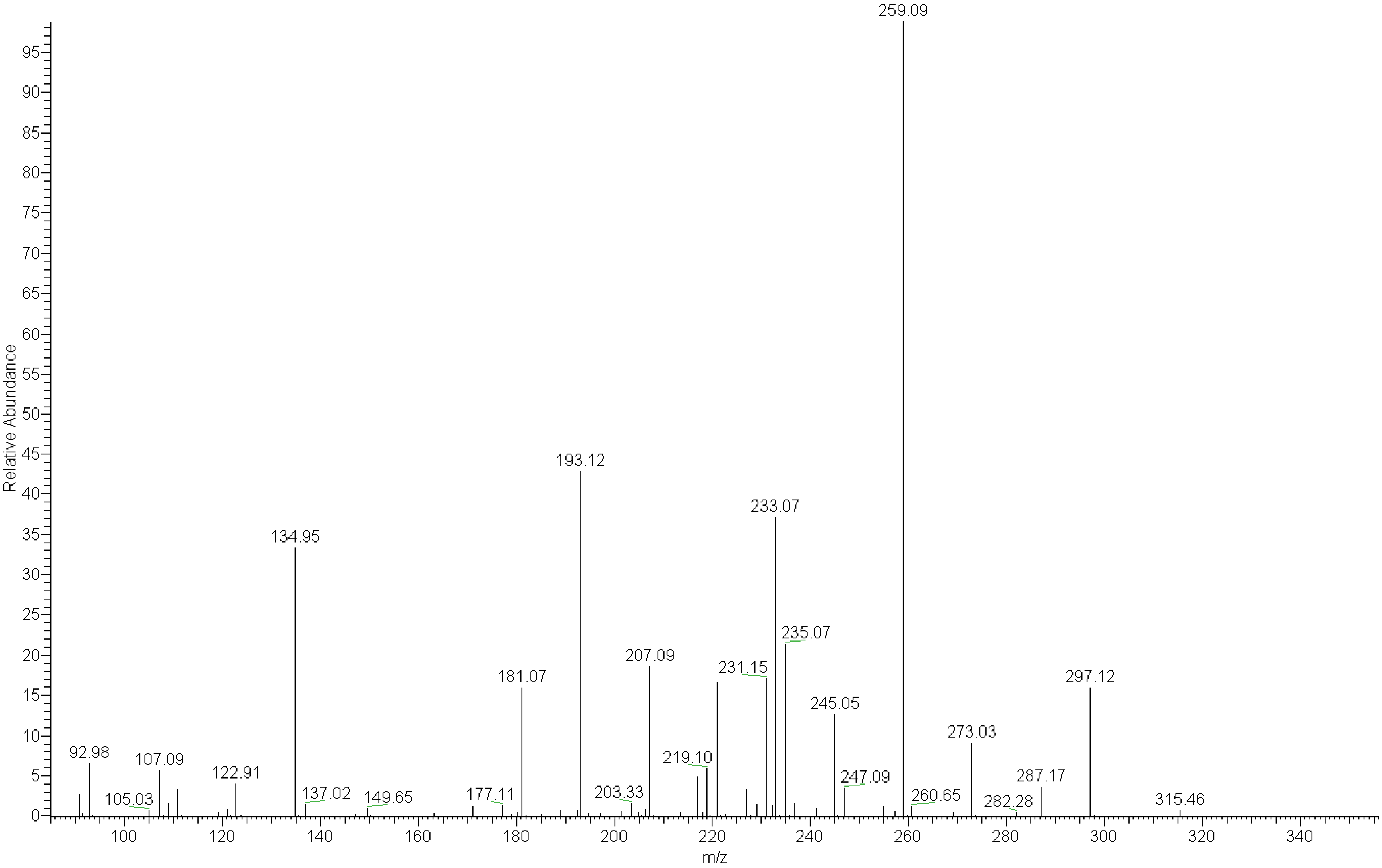

Relative Abundance
m/z
259.09
193.12
233.07
134.95
235.07
207.09
231.15
181.07
297.12
245.05
273.03
219.10
92.98
107.09
122.91
287.17
247.09
105.03
137.02
149.65
177.11
203.33
260.65
282.28
315.46

CBD

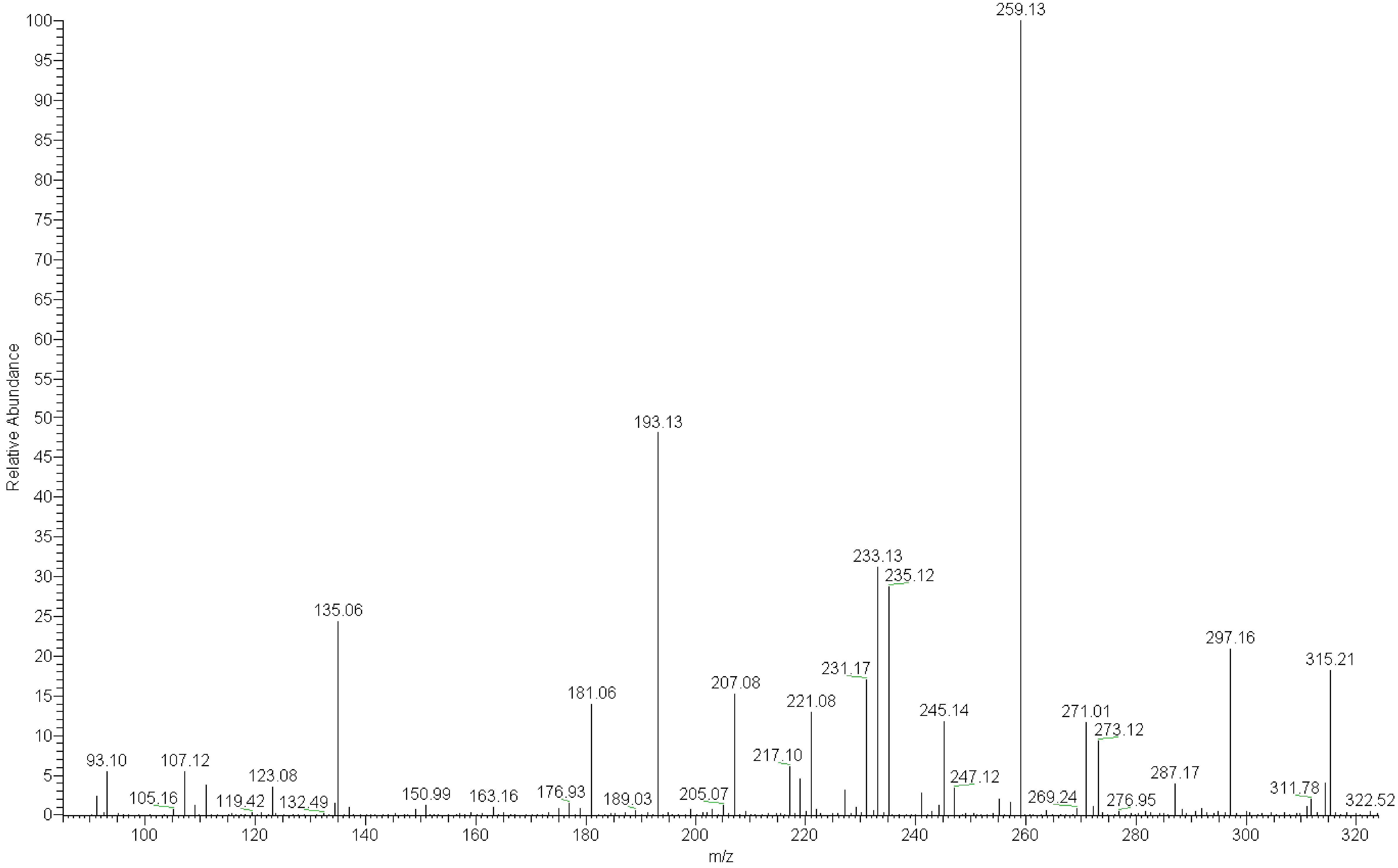
259.13
193.13
233.13
235.12
135.06
297.16
315.21
231.17
207.08
181.06
221.08
245.14
271.01
273.12
217.10
93.10
107.12
123.08
247.12
287.17
311.78
105.16
119.42
132.49
150.99
163.16
176.93
189.03
205.07
269.24
276.95
322.52
Relative Abundance
m/z
